# Unexpected morphological variability in the eggshells of the South American caimans *Caiman latirostris* and *Caiman yacare*

**DOI:** 10.1038/s41598-023-31837-9

**Published:** 2023-03-25

**Authors:** E. Martín Hechenleitner, María V. Fernandez Blanco, Segundo R. Núñez-Campero, Lucas E. Fiorelli, Paula Bona

**Affiliations:** 1 Consejo Nacional de Investigaciones Científicas y Técnicas (CONICET), Godoy Cruz 2290, C1425FQB CABA, Argentina; 2 Centro Regional de Investigaciones Científicas y Transferencia Tecnológica de La Rioja (CRILAR), Provincia de La Rioja, UNLaR, SEGEMAR, UNCa, CONICET, Entre Ríos y Mendoza S/N, 5301 Anillaco, La Rioja Argentina; 3 Instituto de Biología de La Conservación y Paleobiología (IBICOPA) DACEFyN-UNLaR, Av. Gob. Vernet y Apóstol Felipe, 5300 La Rioja, Argentina; 4 División Paleontología Vertebrados, Anexo II Laboratorios del Museo de La Plata, Facultad de Ciencias Naturales y Museo, Universidad Nacional de La Plata, Calles 122 y 60, B1900FWA La Plata, Buenos Aires Argentina

**Keywords:** Zoology, Herpetology, Taxonomy, 3-D reconstruction, X-ray tomography

## Abstract

Eggshell morphology is a valuable indicator of the local conditions within the nests of modern crocodilians and birds. In contrast to these latter, the anatomical structure of the eggshells of most crocodilian species is practically unknown. Here, we provide the first characterization of crocodilian eggshells, using x-ray micro-CT scans. We studied eggshells of *Caiman latirostris* and *Caiman yacare* from various developmental stages that coincide with the beginning of embryonic ossification. The new 3D renderings revealed complex ornamentation, unique among crocodilians, and amphora-shaped pore canals, some of which converge in single pore openings. We also documented a high density of pore canals with a gas diffusion capacity 45 times higher than the average predicted for modern avian eggshells. The external ornamentation and the thickness of the compact layer of the eggshells (i.e. excluding ornamentation) showed ontogenetic and interspecific differences that could be related to nesting materials and nesting areas selected by each species. The shell features described here evidence a greater structural complexity than previously recognized in phylogenetically close, sympatric crocodilian species. Further comprehensive morphological analyses on other modern and fossil crocodilian eggshells using micro-CT technology will shed new light on the evolution of reproductive strategies in this intriguing archosaur clade.

## Introduction

As all oviparous reptiles, crocodilians are particularly vulnerable during their early ontogeny, especially concerning predation and environmental changes affecting their nests^1–3^. The calcified eggshell constitutes the primary physical barrier against external threats and its morphology results from a trade-off between the embryonic need to exchange respiratory gasses with the surrounding nesting environment and provide mechanical and chemical resistance^4^. Associated with underground incubation, which is a common feature for all crocodilians^5^, the eggshells of different species exhibit a few general morphological adaptations, such as relatively high porosity and well-developed external ornamentations^6,7^. These characters show subtle morphological discrepancies between species^4,8–11^, but studies focused on character variation and functional morphology of their eggshells are still scarce^6,12–14^.

The eggs of at least a few crocodilian species, such as *Alligator mississippiensis* and *Caiman latirostris*, are adapted to withstand dissolution of up to 20% of the total thickness of their shells during the incubation process^12,15^. This shell thinning results from a combination of calcium mobilization during embryonic ossification (a process also shared with several archosaurs^16,17^) and the erosion by organic acids during the decomposition of organic matter within mound nests^15^. Along with shell thinning, increased porosity, and structural weakening are expected to occur during the pre-hatching ontogeny^8,11,18^. Although dissolution is evident in the eggshells of several crocodilians, its impact on the morphology of key components, such as the ornamentation and the pore canal system, is still poorly understood.

The pore canal system serves as an indicator of the water balance between the developing embryo and the external nesting environment, as it controls the rate of diffusion of respiratory gasses and water loss^19,20^. This rate, measured under standard conditions of humidity, temperature, and pressure, is known as water vapor conductance (G_H2O_) and aids to establish interspecific comparisons^20^. In general, high G_H2O_ values are associated with nesting environments in which desiccation is not critical, as is the case of several underground-nesting species. Measurements under lab conditions are not always possible but G_H2O_ can also be estimated morphometrically from collection specimens by using pore length and pore cross-sectional area as input data^21–23^. However, this alternative approach is particularly sensitive to variations in the pore geometry^16,21,24–26^. Studies in non-avian dinosaur and bird eggshells using non-destructive, x-ray micro-computed tomography (micro-CT) have shown a remarkable potential to reconstruct, and also quantify, the three-dimensional morphology of different types of pores^27–30^. Among crocodilians, information on pore morphology is scarce and there are no specific studies focused on their variability and its physiological implications. The non-invasive micro-CT technology has not been used yet for assessing porosity nor any other key eggshell features in this clade, although the results in other archosaurs are encouraging.

The present contribution aims to document the three-dimensional anatomy of the eggshells of *C. latirostris* and *Caiman yacare* using micro-CT scans. These closely related species of South American caimans live sympatrically in part of their range, although they differ in the use of their habitat^31^. We explore the interspecific variations and the eggshell morphological changes that occur during the initial phase of embryonic skeletal ossification. Quantitative data regarding shell thickness, pore canals’ geometry, and G_H2O_ in both *Caiman* species is also analyzed. The present findings expand the known morphological variability in the pore canal system and other morphological features of crocodilian eggshells and contribute to recognizing differences between the eggshells of *C. latirostris* and *C. yacare*.

## Results

### *Caiman latirostris*

**Figure 1:**
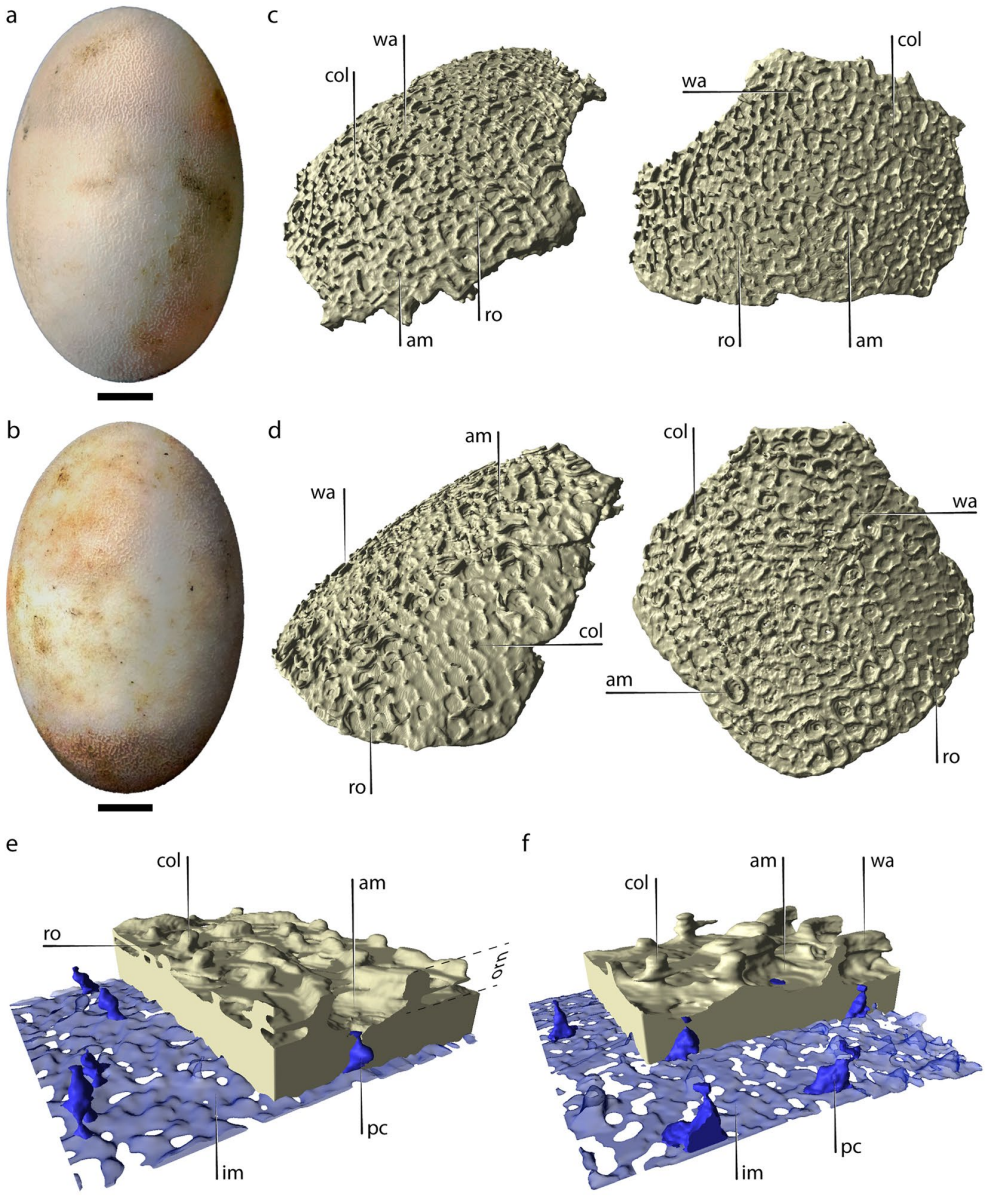
Eggs and eggshells of *C. latirostris*. (**a**) Egg MLP.R.6800-6, CL2 (stage 17/18). (**b**) Egg MLP.R.6800-29, CL6 (stage 21). (**c**) Eggshells of CL2 and (**d**) CL6. (**e**) Detail of the eggshell of CL2 and (**f**) CL6, including pore canals and inner space plus inner shell membrane. Note that all blue-colored structures represent empty spaces. The space reconstructed in transparent blue corresponds to the inner shell membrane plus the space between the latter and the inner shell surface. *am* amphitheater, *col* vertical columns, *im* inner shell membrane, *orn* ornamentation, *pc* pore canal, *ro* roof, *wa* wall. Scale bars in (**a**) and (**b**) equal 10 mm.

All sampled eggs of *C. latirostris* (Supplementary Tables S1, S2) have a single axis of symmetry, with no particular differentiation between both poles (Fig. 1a,b). These oval-shaped structures averaged 69.60 ± 0.85 mm (mean ± SE) in length and 70.70 ± 2.57 cm^3^ in volume (Table 1). The white-colored eggshell of calcite is 656.08 ± 5.55 µm (mean ± SE) thick and presents an organic inner shell membrane with a thickness of 97.21 ± 1.20 µm. The calcitic eggshell is composed of an internal compact layer, 303.65 ± 4.56 µm (mean ± SE) thick, and a conspicuous external ornamentation (Fig. 1c,d). In all stages sampled, this ornamentation represents nearly half of the total shell thickness (shell thickness vs. ornamentation thickness ratio of 0.6) and is composed of vertical stacks of calcite lamellae, which often surround excavations of the shell surface (Fig. 1e,f). Moreover, we have observed that pore canals open up externally in the center of these surficial depressions (Supplementary Fig. S1). Vertical columns of the ornamentation are circular to oval in cross-section and sometimes extend laterally, forming rather long walls with different orientations (Fig. 1c,d). These columns and walls bordering depressions that contain pore openings define a characteristic pattern that reminds an amphitheater (Fig. 1c–f). The height of these columns and walls is homogeneous across the surface of each egg but varies between specimens (see Supplementary Figs. S2–S13 and Supplementary Renderings S1). In turn, columns often project themselves laterally forming thin lateral extensions, parallel to the eggshell surface (i.e. bridges), which join adjacent columns and walls (Fig. 1e,f). Each column can develop several of these flat bridges, one above the other, defining up to three different levels. In our sample, it is common to observe bridges connecting several columns and walls, creating massive roofs that can locally cover a broad shell area. Bridges are occasionally situated above some pore openings.

**Table 1:** Measurements and estimations of egg and eggshell characters in *C. latirostris* and *C. yacare*.

		*Caiman latirostris*	*Caiman yacare*
Eggs	n	6	6
Egg length (mm)	Mean ± SE	69.60 ± 0.85	68.20 ± 0.73
Egg volume (cm^3^)	Mean ± SE	70.70 ± 2.57	81.21 ± 2.49
Egghell thickness (µm)	Mean ± SE	656.08 ± 5.55	617.87 ± 14.17
Compact layer thickness (µm)	Mean ± SE	303.65 ± 4.56	246.10 ± 1.71
Ornamentation ratio		0.5	0.6
Inner shell membrane (µm)	Mean ± SE	97.2 ± 1.20	96.71 ± 1.45
Pore canals	n	180	199
Pore density (pores/cm^2^)	Mean ± SE	47.62 ± 3.55	52.65 ± 3.73
Pore geometry (%)	Amphora	88.33	72.36
	Others	11.67	27.64
Pore lateral connections (%)	Simple	76.67	89.45
	Convergent	23.33	10.55
Single pore GH2O (µg/day.Torr)	Mean ± SE	71.59 ± 3.44	72.70 ± 2.81

See also Supplementary Table S2.

The pore canals varied in size and shape regardless of ontogeny, and some of them coalesce half of their path through the outer shell surface. We calculated an average of 47.62 pores/cm^2^ and 88.3% of them (n = 180) have a broader cross-sectional area in the middle of their path. They open externally in a funnel-shaped aperture, defining an amphora-shaped profile (Fig. 1e,f). The internal openings, at the opposite end, are usually larger than the external ones. The rest of the pores vary within a gradient between amphora-shaped and nearly straight, encompassing an array of pores that do not have a noticeable broader cross-section in the middle of their path. They represented less than 15% of the sample, including uniformly thin pores, pores with wide internal openings, and others whose diameter reduces towards the outside (cone-shaped). We also observed that up to three pores (23.3% in abundance) can converge in the middle of their path and reach the outer shell surface through a single opening (e.g. Fig. 1e).

**Figure 2:**
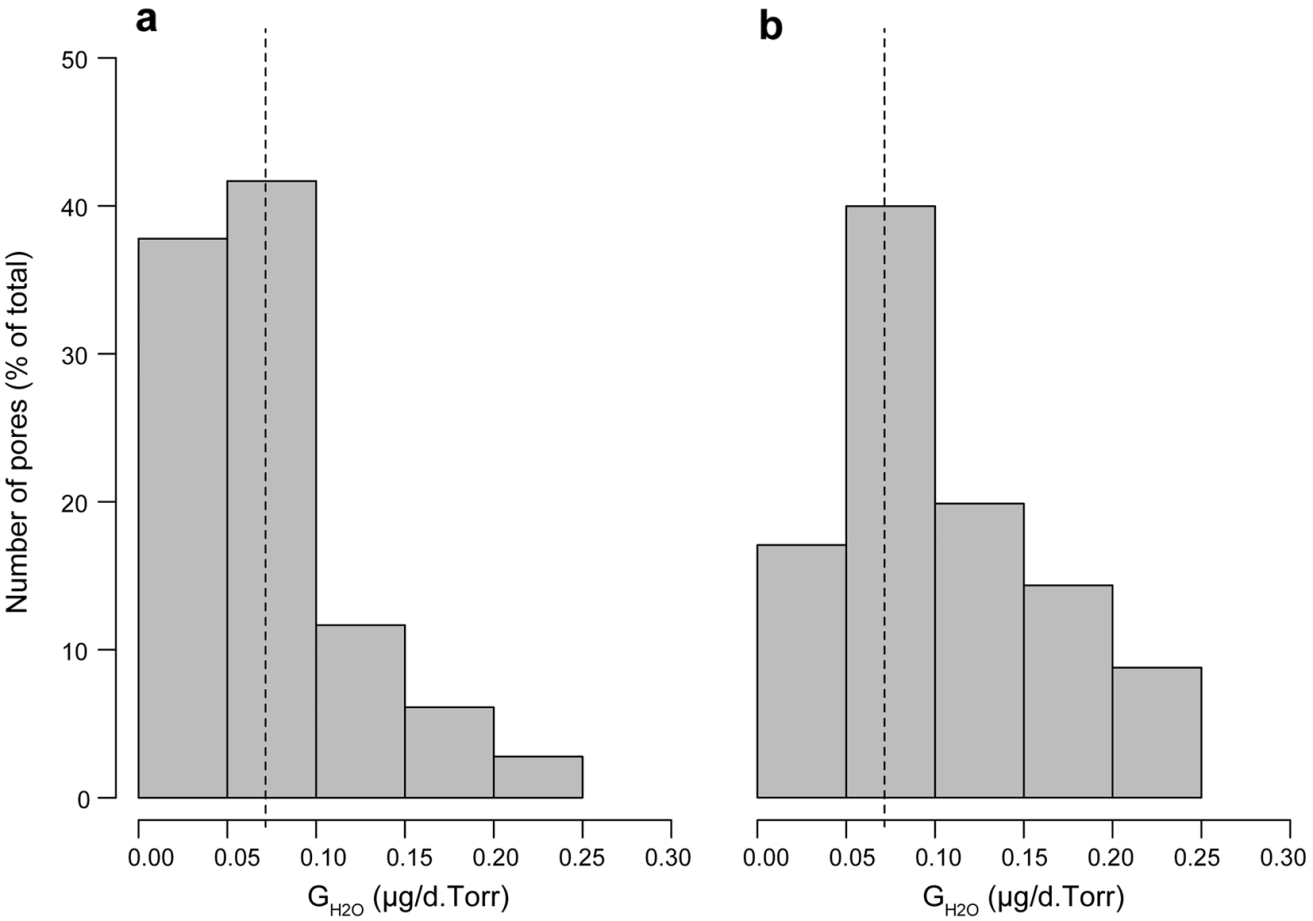
G_H2O_ per pore in *Caiman latirostris*. (**a**) Distribution for 180 single pores. (**b**) Contribution of each range to the total G_H2O_ of the sample. The dashed line corresponds to the average single pore G_H2O_, which equals 71.59 µg/d.Torr.

Comprising all the sampled stages, the average single pore G_H2O_ was 71.59 ± 3.44 µg/d.Torr (mean ± SE), and nearly 80% of the pores (n = 180) showed a G_H2O_ below 100 µg/d.Torr (Supplementary Table S3). The pores with G_H2O_ values lower than 50 µg/d.Torr were almost as abundant as those whose G_H2O_ falls within the range 50–100 µg/d.Torr (Fig. 2a), but their contribution to the total G_H2O_ was less than 20% (Fig. 2b). In contrast, the pores within the interval 100–150 µg/d.Torr are relatively few (~ 12%), but their contribution is comparatively larger than those from the < 50 µg/d.Torr interval.

As development advanced, we observed some structural changes in the shell. In the first two stages sampled (CL1 and CL2) the ornamentation is well-developed and the roofs are remarkable (Fig. 1c,e). Although these roofs are still present in the last stage analyzed (CL6), their abundance and lateral coverage decrease considerably throughout ontogeny (Fig. 1d,f). However, some remnants of these ornaments are barely visible in a few sectors of CL5 and CL6 (Supplementary Figs. S9–S12). As they disappear, the ornamentation reduces to isolated columns and walls of varying lengths. Additionally, the compact layer of the shell (i.e. without ornamentation) was thicker in the first stage analyzed and progressively thinner in the following stages (− 12.6x + 335; R^2^ = 0.75), and we found significant differences between a few stages (F_(5, 174)_ = 31.52, p < 0.001). It is worth noting that the thickness of the ornamentation did not vary significantly ontogenetically within the analyzed sample.

### *Caiman yacare*

**Figure 3:**
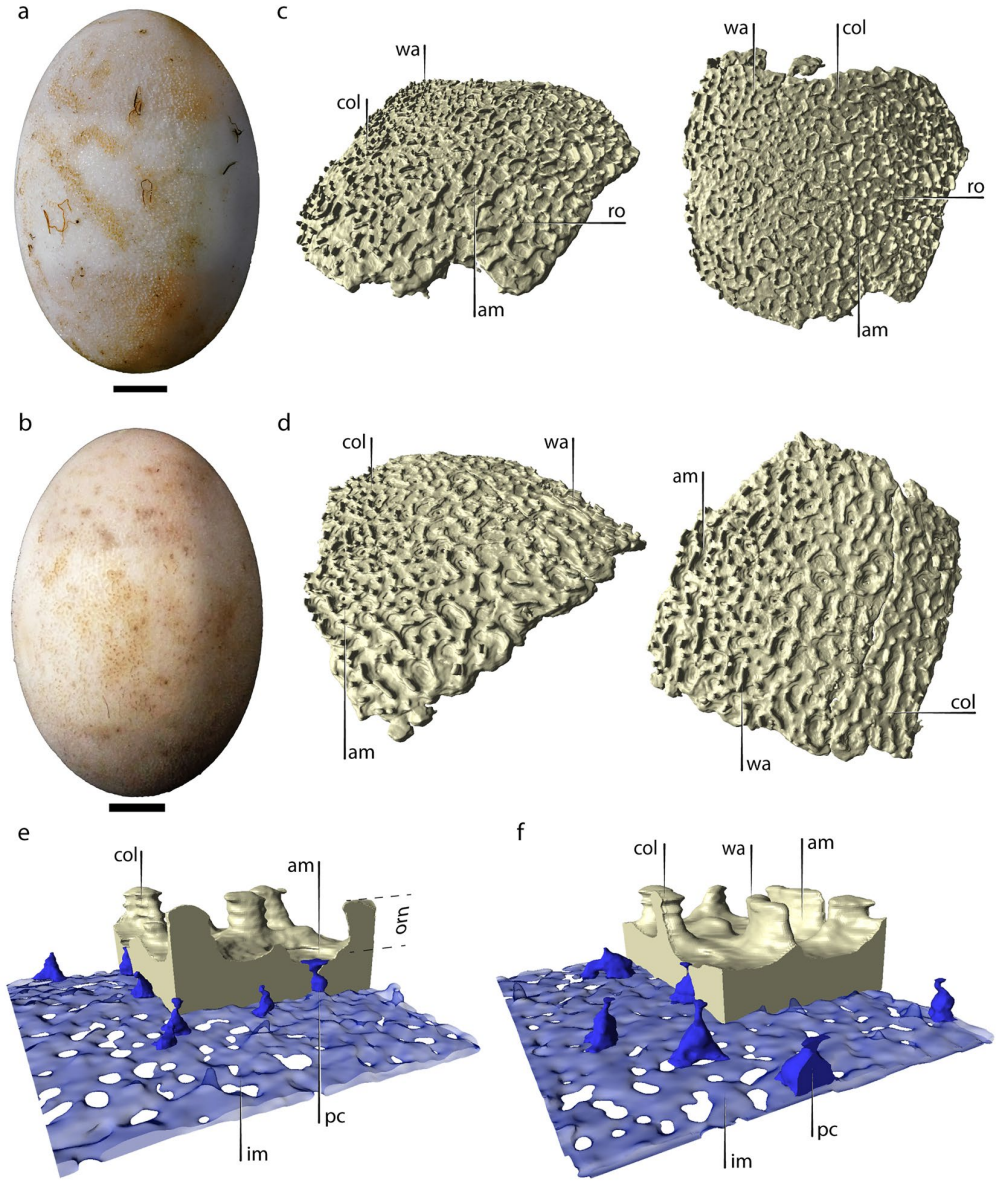
Eggs and eggshells of *C. yacare*. (**a**) Egg MLP.R.6800-39, CY2 (stage 18). (**b**) Egg MLP.R.6800-66, CY6 (stage 23). (**c**) Eggshells of CY2 and (**d**) CY6. (**e**) Detail of the eggshell of CY2 and (**f**) CY6, including pore canals and inner space plus inner shell membrane. Note that all blue-colored structures represent empty spaces. The space reconstructed in transparent blue corresponds to the inner shell membrane plus the space between the latter and the inner shell surface. *am* amphitheater, *col* vertical columns, *im* inner shell membrane, *orn* ornamentation, *pc* pore canal, *ro* roof, *wa* wall. Scale bars in (**a**) and (**b**) equal 10 mm.

All sampled eggs of *C. yacare* (Supplementary Tables S1, S2) are also white and oval, averaging 68.20 ± 0.73 mm (mean ± SE) in length and 81.21 ± 2.49 cm^3^ in volume (Fig. 3a,b). The eggshells of the six stages measured 617.87 ± 14.17 µm (mean ± SE) thick. Their compact region was 246.10 ± 1.71 µm (mean ± SE) thick, whereas the inner shell membrane was 96.71 ± 1.45 µm. The outer surface of the eggshells is irregular, with conspicuous ornamentation that is thicker than the compact layer (ornamentation vs. compact layer thickness ratio of 0.6). Regarding its architecture, the ornamentation is similar to but simpler than that of *C. latirostris* (Supplementary Figs. S13–S24 and Supplementary Renderings S2). Columns and walls are abundant (Fig. 3c,d), although they are reduced to low ridges in some samples of intermediate stages, such as CY3 (stage 19) and CY4 (stage 20). The most noticeable difference from *C. latirostris* is the scarcity of bridges and roofs connecting columns and walls (Fig. 3c–f), particularly in CY1 (stage 17–18), CY2 (stage 18), and CY5 (stage 22). The lateral expansions parallel to the shell surface are also less frequent than in *C. latirostris*. Exceptionally, some specimens such as CY2 show a similar ornamentation pattern to that observed in equivalent ontogenetic stages of *C. latirostris*. The eggshell of some specimens (e.g. CY3, CY4, and CY6), show external surfaces partially devoid of ornamentation, with few mound-shaped, low columns and bridges (see Supplementary Figs. S13–S24).

The pore canals in all stages analyzed of *C. yacare* are similar in size and shape to those of *C. latirostris*. We estimated 52.65 pores/cm^2^ for the whole shell sample, and most of the pores are amphora-shaped (72.4% in abundance; n = 199). We also documented convergent pores but fewer than in *C. latirostris* (Table 1; Supplementary Figs. S13–S24).

**Figure 4:**
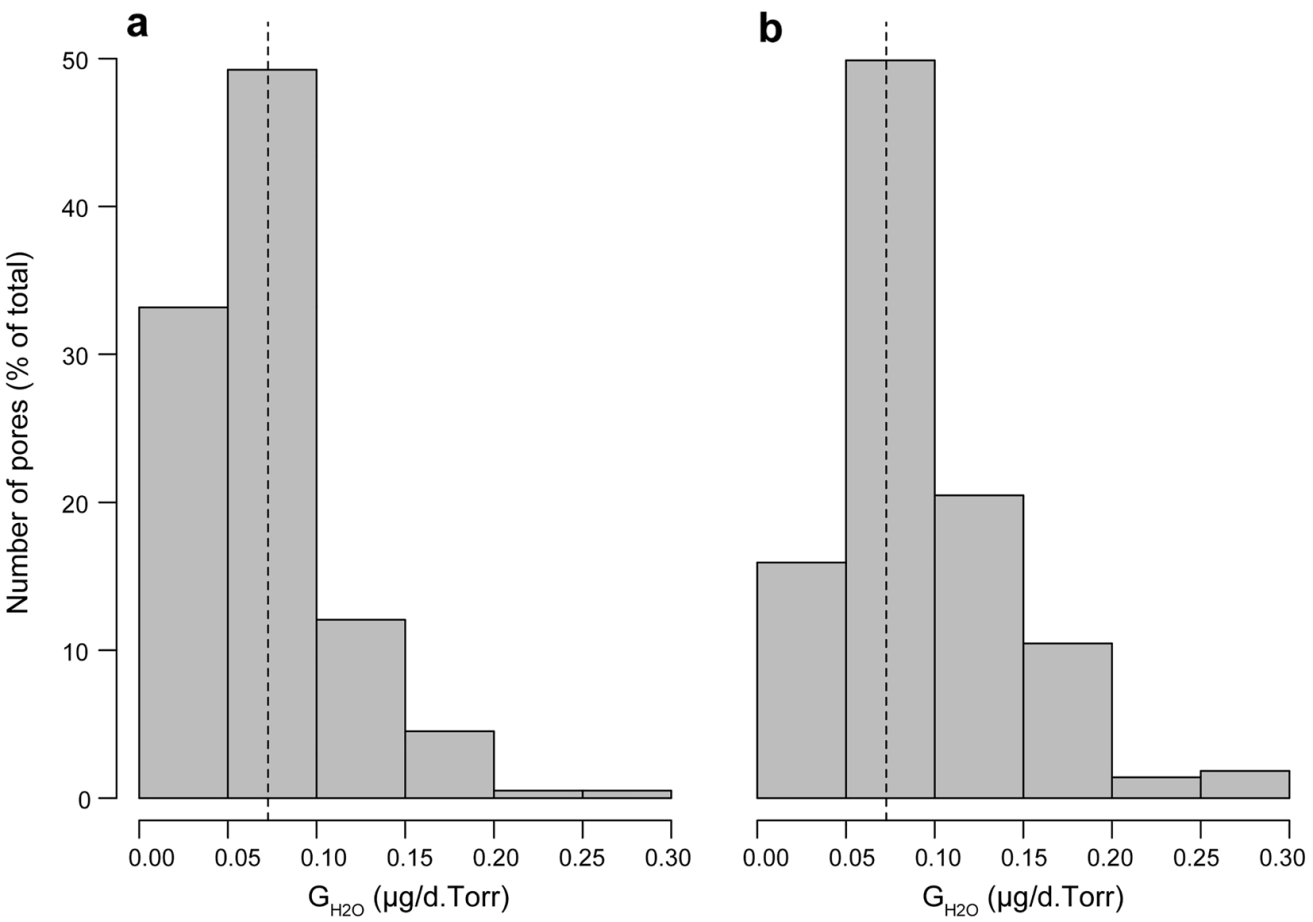
Single pore G_H2O_ in *Caiman yacare*. (**a**) Distribution for 199 single pores. (**b**) Contribution of each range to the total G_H2O_ of the sample. The dashed line corresponds to the average single pore G_H2O_, which equals 72.70 µg/d.Torr.

The estimated single pore G_H2O_ for all the eggshell samples of *C. yacare* was 72.70 ± 2.81 µg/d.Torr (mean ± SE) (Supplementary Table S4). As in *C. latirostris*, most of the pore canals have estimated G_H2O_ below 100 µg/d.Torr (Fig. 4a). Moreover, the pores in the range of 100–150 µg/d.Torr were also substantially less but contributed more to the total G_H2O_ than those with G_H2O_ below 50 µg/d.Torr (Fig. 4b).

Some ontogenetic morphological variations among specimens of *C. yacare* are evident but do not follow a clear trend. The ornamentation shows more walls and bridges in the more developed CY2 (stage 18) than in CY1 (stage 17–18) and is also thicker and more complex than in CY3 (stage 19) and CY5 (stage 22) (see Supplementary Figs. S13–S24). Moreover, differences in the thickness of the compact layer of the shells are statistically significant among some specimens (F_(5, 174)_ = 3.73, *p* = 0.003). However, these variations did not show any specific tendency throughout the ontogeny.

### General comparisons

**Figure 5:**
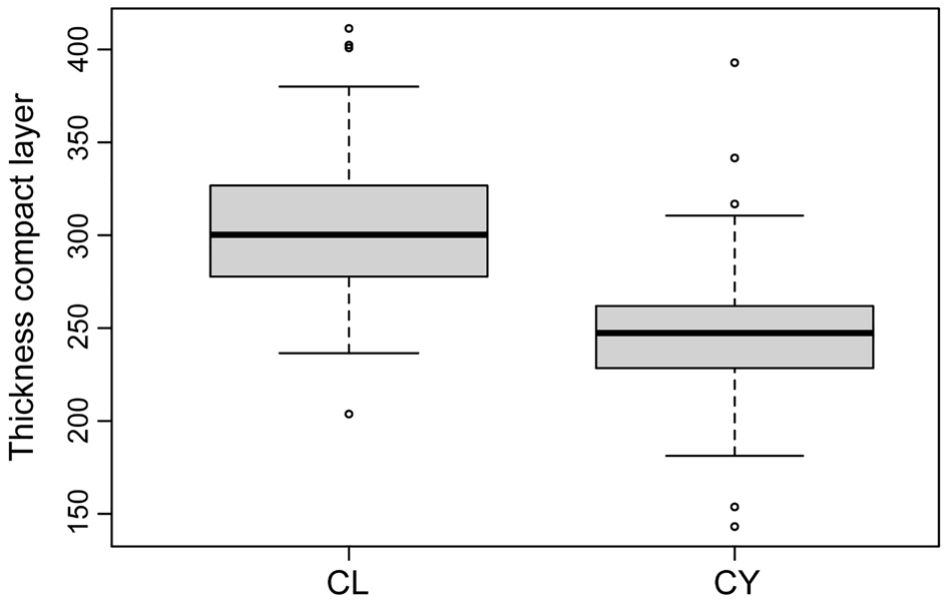
Comparison of the thickness of the compact region of the eggshell in *C. latirostris* and *C. yacare*. Mean values are 303.65 ± 4.56 µm (mean ± SE) and 246.10 ± 1.71 µm, respectively.

Among the sampled specimens, the compact layer of the shells of *C. latirostris* is much thicker than in *C. yacare* (χ^2^ = 174.07, *df* = 1, *p* < 0.001; Fig. 5). In contrast, the ornamentation of the latter species is proportionally thicker, although it does not show as many lateral connections (i.e., bridges) as in *C. latirostris*. We observed that some regions of the eggshells of both species have columns and walls aligned with flanking narrow valleys, whose predominant path is parallel to the major axis of the egg (Figs. 1c, 3c,d).

The pore canals of both species share similar pore densities and single pore G_H2O_ values along the ontogeny (Supplementary Tables S2–S4). However, sampled specimens of *C. latirostris* have proportionally more amphora-shaped pores and more convergent pores than those of *C. yacare*.

## Discussion

In general, the eggs of *C. latirostris* and *C. yacare* are similar in shape and size, but the micro-CT scans reveal some microanatomical differences between species. The eggs of both species are symmetrical and their size is intermediate to other extant crocodilians, such as *A. mississippiensis* and *Crocodylus mindorensis*^6^. Marzola et al.^6^ reported an average shell thickness for these latter species of 0.53 and 0.43 mm, respectively, whereas the thicknesses (without ornamentation) in *C. latirostris* and *C. yacare* are just 0.30 and 0.25 mm, respectively (Tables 1, 2). It is unclear if such discrepancies correspond to different shell microstructural arrangements in different taxa. However, considering the similar egg size in both *Caiman* species, the present data suggest a greater mechanical resistance for the shells of *C. latirostris*, which selects places more resistant to flooding (i.e. forest^32^), compared to those of *C. yacare*, which prefers nesting in heavily vegetated water bodies (i.e. floating vegetation^32^). Alternatively, thicker eggshells may represent an adaptation to resist some external chemical degradation. It would be interesting to determine whether the differences in the composition of the microbial biota of each environment (forest vs. floating vegetation) may generate particular conditions with local effects on the rate of shell dissolution.

**Table 2:** Morphological features that characterize the eggshells of some alligatorids.

	Eggshell thickness (mm)	Ornamentation thickness/compact layer thickness ratio	Ornamentation morphology	Pore morphology	Pore lateral connections	Pore density (cm^2^)	Source
*C. latirostris*	0.66 (0.30)	0.5	Well-developed lateral connections, including large roofs in earlier stages	Amphora	Present	48	This study
*C. yacare*	0.62 (0.25)	0.6	Multiple bridges, only in early stages	Amphora	Present	53	This study
*A. mississippiensis*	0.53	?	Low, elongate ridges; sometimes ramified	Straight—amphora?	?	5	Marzola et al.^6^; Ferguson^8^

*Values in parenthesis correspond to thickness without considering ornamentation (i.e. compact layer). Note that although *C. latirostris* and *C. yacare* share columns and walls in there ornamentations, the development of lateral interconnections is greater *C. latirostris*.

The thickness of the ornamentation and its structural complexity are distinctive features of *Caiman* eggshells. The external ornamentation in archosaur eggshells has been largely associated with ground nesting^6,33–36^ and may have evolved independently in different lineages^37^. While just a few birds exhibit subtle external ornamentation^38–41^, in several clades of non-avian dinosaurs this feature frequently exhibits a greater development^33,35,42–48^. Among crocodilians, ornamentation is also frequent and morphologically variable, from subtle expressions such as the ridges with shallow and scattered pits described in *Cr. mindorensis*^6^ to the anastomosed pattern observed in *A. mississippiensis*^6^. All these ornamentations reach little development compared to the structures observed in *C. latirostris* and *C. yacare*, which represent as much as 50–60% of the total shell thickness. In *C. latirostris*, the lateral interconnections among columns and walls are comparatively more abundant than in *C. yacare* and develop patches of complete coverage (i.e. roofs) in many areas of the external egg surface. Such differences, as well as a thicker compact layer of the eggshell in *C. latirostris*, demonstrate the potential of shell morphological characters to identify fragmentary material at a specific level (Table 2). On the other hand, the present results add to a list of previous studies that suggest caution when considering the systematic relevance of traits with marked intraspecific variability, such as egg size^4,49^. Moreover, additional morphological variations result from pre and post mortem dissolution processes, as is frequently seen in the fossil record^6,23^. For a better understanding of such changes, it would be useful to extend the sampling to other living crocodilian species, especially to those that have never been studied under any methodological approach.

The new micro-CT renderings revealed microstructural shell alterations through the embryonic development of *C. latirostris* and *C. yacare*. Ferguson^15^ demonstrated strong external acidic dissolution of the eggshells coupled with internal dissolution mediated by calcium mobilization during the development of *A. mississippiensis*. Simoncini et al.^12^ also documented a 20% thinning in the shells of *C. latirostris* throughout the whole incubation process, although whether this latter corresponds to intrinsic or extrinsic dissolution or a combination of both, remains unclear. Although our sampling only covered a fraction of the incubation process (stages 15 to 21 in *C. latirostris* and 17–18 to 23 in *C. yacare*), it was enough to detect a significant thinning in the compact layer of the eggshells of *C. latirostris*. Such thickness reduction overlaps with the onset of skeletal ossification in both species, which occurs between stages 17–18 and 23^50,51^. Consequently, mobilization of calcium from the eggshell would be expected. Furthermore, the reconstructions show a gradual loss of connections between columns of the ornamentation (i.e. roofs and bridges), a rounding on all structures (e.g. CL1 compared to CL4), and a widening of the periphery of the pore openings. Thus, an important component of external dissolution is indirectly evidenced in both *Caiman* species in this study.

Interestingly, the present results suggest that the external dissolution has minimally affected the radial extension of the ornamental columns and walls, as they do not appear broken at a first-hand examination or in the micro-CT scans. These structures could hold the gap between the pore openings and the nesting material throughout most of the incubation process. In this way, the columns could be functionally similar to the ornaments described for some non-avian dinosaurs that also incubated underground. In the gigantic titanosaur sauropods, the densely packed nodules of the ornamentation could have mitigated the acid erosion of the environment and prevented the obstruction of the external pore openings^17,36^. The lateral interconnections between columns and walls in *C. latirostris* and *C. yacare* are extremely thin radially, but widen tangentially to the egg surface, maximizing the contact area with the nesting environment. These tangential extensions do not seem to make a substantial contribution in terms of strength, as they are extremely brittle when specimens are handled. However, they could enhance the capacity of the shell to retard external dissolution, whereas the columns and walls remain for a longer period, retaining an air cushion that would facilitate a homogeneous distribution of respiratory gasses throughout much of the egg surface. This would contribute to enduring regional hypoxia^52^. Recently, Cedillo-Leal et al.^14^ proposed that the ornamentation of *C. latirostris* increases the chances of embryonic survival during momentary flooding episodes by trapping air bubbles near the pore canal openings. Consistently, all these observations suggest that the ornamentation of *Caiman* contributes to retaining the nesting sediment apart from the shell surface, facilitating the surficial circulation of respiratory gasses, and minimizing the effects of extrinsic dissolution.

The pore canals in *C. latirostris* and *C. yacare* eggshells are abundant, even compared to some of their living counterparts. High pore densities are frequent in underground-incubating species, such as extant crocodilians. The eggs of *A. mississippiensis* barely have 5 pores/cm^2^ on average, whereas the pore counts in *Cr. mindorensis* and *Paleosuchus palpebrosus* are as high as 21 and 22 pores/cm^2^, respectively^6^. Although the pore density varies regionally within a single egg, the pore counts in our samples of *C. latirostris* and *C. yacare* reached ~ 48 and ~ 53 pores/cm^2^, a much higher amount than expected for eggs of a similar size. Besides, the numerous pores arranged along longitudinal depressions flanked by the columns and walls that make up the ornamentation could represent lines of weakness that may facilitate hatching.

The amphora-shaped pore morphotype observed in the *Caiman* species differs markedly from that described for other archosaur species, such as birds and non-avian dinosaurs in general. Most bird eggshells typically exhibit simple trumpet-shaped pore canals whose diameter increases from the inside out^27,53^. This shape determines that much of the resistance to gas diffusion occurs near the internal opening of the pores at the base of the shell^24,25^. In most birds, the intrinsic degradation during the embryonic ossification phase is minimal and does not affect the morphology of the pore canal system^54^. However, in precocial species, such as megapodes, the thinning process eliminates the narrowest fraction at the base of the pore canals, increasing their gas diffusion potential^16^. This late thinning coincides with an increase in the embryonic metabolic rate before hatching^16^. In contrast to this condition, the amphora-shaped pores of *C. latirostris* and *C. yacare* have narrower cross-sections near the outer shell surface (Figs. 1e,f, 2e,f). Although the sampled ontogenetic interval did not reveal appreciable differences in G_H2O_ in any of the *Caiman* species, the particular geometry of their pore canals suggests that external degradation of the eggshell towards the end of the incubation period^12^ could increase their porosity. The loss of the narrowest fraction of the pores, as a result of external dissolution, would be essential in driving metabolic activity toward the end of their embryonic development. Moreover, the thinning process could facilitate hatching, as seen in *A. mississippiensis*^15^. The amphora-shaped pore morphotype, therefore, may represent an adaptive novelty among archosaurs with a significant physiological impact on species that tolerate considerable external degradation of their eggshells.

The amphora pores ensure high G_H2O_ and could be a common morphotype among crocodilians. In addition to being abundant, the amphora-pores described here are proportionally wider than those of other archosaur groups, such as birds^53^. For example, the pore canals in *Gallus gallus*, whose path length is similar to that of the *Caiman* pores, have a G_H2O_ of around 1.5 µg/d.Torr^25,55^. However, the pores of *C. latirostris* and *C. yacare*, with larger cross-sectional areas, have much higher G_H2O_ values (Figs. 2, 4; Table 1). Although quantitative data on other crocodilians is still unavailable, the amphora-shaped pores are similar in size and shape to those of some related species. Some specimens of *Cr. mindorensis* (Marzola et al.^6^, Fig. 8) and *A. mississippiensis* (Ferguson^8^, plate II, a; Wink and Elsey^56^, Fig. 3a) may have an equivalent geometry. Ferguson^8^ described the pores of *A. mississippiensis* as “golf tee (-shaped) with the campanulate orifice outermost”, which suggests that these crocodilians may display an array of pore morphotypes as seen in *C. latirostris* and *C. yacare*. Although there is not enough data to determine whether the amphora morphotype is also the dominant pattern for other crocodilians, the similarities with the pores of *Caiman* suggest high single pore G_H2O_.

The micro-CT scans also revealed the first occurrence of laterally-connected pore canals in crocodilian eggshells. Lateral connections between pore canals are frequent among ground-nesting saurischian dinosaurs. Branching prevails in the pores of sauropod dinosaur eggshells, especially in those that are proportionally thick^17,23,28^. Among modern birds, the Australian megapode *Alectura lathami*, which incubates in mounds of decaying vegetation, produces eggs with high densities of Y-shaped pore canals, most of which develop lateral connections with nearby pores^40^. Their pore system facilitates the diffusion of gasses in the humid atmosphere of their mound nests^40^. A more complex pattern with multiple branching is well-known in the thick-shelled eggs of *Struthio camelus*^29^, and other ratites as well^53,57,58^. However, in both birds and non-avian dinosaurs, branches always multiply outwards^23,29,53,59–63^, which contrasts with what was observed here in *Caiman*. Many pores of *C. latirostris* and *C. yacare* are composed of up to three pore canals that converge half the way through the outer shell surface. These interconnections in the pore network of *C. latirostris* and *C. yacare* further support the hypothesis of a potential advantage in gas exchange performance in oxygen-depleted and highly humid nesting environments.

## Conclusion

The present micro-CT scans reveal a more intricate eggshell microstructure in *Caiman* than previously considered. The external ornamentation presents a general architecture and a thickness characteristic of the genus, but also shows subtle differences between species, such as the more prominent development of lateral interconnections between columns and walls in *C. latirostris*. This, coupled with a greater thickness of the compact layer awards stronger mechanical or chemical properties for the eggshell of *C. latirostris*, which also undergoes a gradual structural simplification of the ornamentation throughout ontogeny. The ornamentation could increase the efficiency of the pore canal system, avoiding pore clogging and facilitating the circulation of gasses over the external surface of the shell. On the other hand, the amphora-shaped pores and the pores that converge towards a single external opening confer a high G_H2O_ to the eggshells of *C. latirostris* and *C. yacare*. Further morphological, physiological, and ecological studies are still required to determine which variables have been significant drivers for eggshell morphological evolution among crocodilians.

## Material and methods

### Egg samples

We studied eggshells of two extant *Caiman* species inhabiting Argentina: *C. latirostris* and *C. yacare*. The eggs are housed in the herpetological collection of the Museo de La Plata (MLP) under the collection number MLP.R.6800 (eggs of different stages were renamed for the sake of simplicity; see Supplementary Table S1). With the permission of the Herpetology Division of MLP, we scanned the shells of six eggs of each species.

The eggs were originally harvested from two different types of nesting habitats during the summer of 2018 in Chaco province (Argentina): forest in the case of *C. latirostris* and floating vegetation for *C. yacare*^32,64^. We used material from one nest per species. Eggs were artificially incubated in their original substrate, under constant conditions of humidity (95%) and temperature (30 ± 1 °C). Embryonic stages, from day 15–39 of incubation, were established using the criterion of Iungman et al.^65^. Information regarding the ossification degree of the embryonic skeleton was obtained from previous studies^50,51^.

### Micro-CT scan

We analyzed a single eggshell fragment of around 100 mm^2^ from the shoulder (i.e. the transitional region between the equator and one of the poles) of each egg, totalizing 12 samples.

A scan of the whole eggshell sample was carried out at YPF-Tecnología, La Plata, Buenos Aires, Argentina. The analysis was performed on a Bruker SkyScan 1172 micro-CT scanner, at a voxel resolution of 13.93 µm. Projection images were obtained at rotation steps of 0.3°, applying 110 kV and 42 µA.

We analyzed the micro-CT data set with the software Avizo (VSG|FEI Visualization Sciences Group, Thermo Fisher Scientific Inc.). We developed a specific protocol to avoid curvature effects in the calculation of pore cross-sectional areas. Although it is ideal to scan the largest shell area possible, the effect of curvature prevents taking orthogonal measurements throughout the entire sample. Therefore, we resampled six sectors within each shell fragment, on which we measured multiple cross-sectional areas along the pore canals’ path (e.g. Supplementary Fig. S1). Pore measurements summarized in Supplementary Tables S3, S4 were obtained by conducting a label analysis on the samples with each voxel being assigned to whether a pore canal, eggshell or exterior, based on the attenuation of the x-ray beam.

### Morphometric G_H2O_

For the morphometric calculation of the single pore G_H2O_, we applied the equations from Tøien et al.^24^ and Hechenleitner et al.^28^, which are derived from the proposal of Ar et al.^20^. Pore G_H2O_ was obtained from its reciprocal, the diffusive resistance (see Tøien et al.^24^). The equation based on Fick’s first law of gas diffusion:

$${R}_{s}=k\frac{{L}_{s}}{{A}_{n}},$$

where *R*_*s*_ is the resistance of each pore segment, *A*_*n*_ is the area (mm^2^) of each pore segment and *L*_*s*_ corresponds to its length (mm). The constant *k*, which has a value of 446.6, corresponds to the diffusion of water vapor in day/Torr.mg, at a standard temperature of 25 °C.

Additionally, we analyzed the aperture effect of internal and external pore openings through the following equation:

$${R}_{ap}=k\frac{1}{4\sqrt{\frac{{A}_{p}}{\pi }}},$$

where *R*_*ap*_ is the resistance of each pore opening, *A*_*ap*_ is the area (mm^2^) of each aperture and the constant *k* is the same as the one used in the previous equation.

The pore’s G_H2O_ is obtained as follows:

$${R}_{diff}=\sum^{n}{R}_{s}+\sum^{n}{R}_{ap}=\frac{1}{{G}_{p}},$$

where *R*_*diff*_ (in day.Torr/mg) is the total resistance of the pore, and *G*_*p*_ is the reciprocal single pore G_H2O_.

### Statistical methods

The statistical analyses were performed in R^66^. As the data did not attain the assumption for parametric analysis, it was analyzed with the non-parametric Kruskal–Wallis test to verify statistical differences between thicknesses of the compact layer of the eggshell (i.e. without ornamentation) for both *C. latirostris* and *C. yacare*. We applied an ANOVA model for the intra-specific analyses of the thickness of the compact layer of the eggshell and G_H2O_ comparison. Graphics were generated using the ggplot2 package^67^.

## Supplementary Information

Supplementary Information 1.

Supplementary Information 2.

Supplementary Information 3.

Supplementary Table S4.

Supplementary Table S5.

## Data Availability

All measurements, as well as the 3D reconstructions, are included in the main text and as supplementary files. Materials are available from the authors upon reasonable request.

## References

[1] Larriera A, Piña CI (2000). *Caiman latirostris* (Broad-snouted Caiman) nest predation: Does low rainfall facilitate predator access?. Herpetol. Nat. Hist..

[2] Doody JS, Freedberg S, Keogh JS (2009). Communal egg-laying in reptiles and amphibians: Evolutionary patterns and hypotheses. Q. Rev. Biol..

[3] Somaweera R, Brien M, Shine R (2013). The role of predation in shaping crocodilian natural history. Herpetol. Monogr..

[4] Ferguson MWJ, Gans C, Billett F, Maderson PFA (1985). Reproductive biology and embryology of the crocodilians. Biology of the Reptilia.

[5] Brazaitis P, Watanabe ME (2011). Crocodilian behaviour: A window to dinosaur behaviour?. Hist. Biol..

[6] Marzola M, Russo J, Mateus O (2015). Identification and comparison of modern and fossil crocodilian eggs and eggshell structures. Hist. Biol..

[7] Lutz PL, Bentley TB, Harrison KE, Marszalek DS (1980). Oxygen and water vapour conductance in the shell and shell membrane of the American crocodile egg. Comp. Biochem. Physiol. A.

[8] Ferguson MWJ (1982). The structure and composition of the eggshell and embryonic membranes of *Alligator*
*mississippiensis*. Trans. Zool. Soc. London.

[9] Schleich H, Kästle W (1988). Reptile Egg-Shells SEM Atlas.

[10] Deeming DC, Ferguson MWJ (1990). Methods for the determination of physical characteristics of eggs of *Alligator mississippiensis*: A comparison with other crocodilian and avian eggs. Herpetol. J..

[11] Wink CS, Elsey RM, Bouvier M (1990). Porosity of eggshells from wild and captive, pen-reared alligators (*Alligator*
*mississippiensis*). J. Morphol..

[12] Simoncini MS, Fernández MS, Iungman J (2014). Cambios estructurales en cáscaras de huevos de *Caiman latirostris*. Rev. Mex. Biodivers..

[13] Fernández MS, Simoncini MS, Dyke G (2013). Irregularly calcified eggs and eggshells of *Caiman*
*latirostris* (Alligatoridae: Crocodylia). Naturwissenschaften.

[14] Cedillo-Leal C (2017). Eggshell structure in *Caiman*
*latirostris* eggs improves embryo survival during nest inundation. Proc. R. Soc. B.

[15] Ferguson MWJ (1981). Extrinsic microbial degradation of the alligator eggshell. Science.

[16] Booth DT, Seymour RS (1987). Effect of eggshell thinning on water vapor conductance of malleefowl eggs. Condor.

[17] Grellet-Tinner G, Fiorelli LE (2010). A new Argentinean nesting site showing neosauropod dinosaur reproduction in a Cretaceous hydrothermal environment. Nat. Commun..

[18] Jenkins NK (1975). Chemical composition of the eggs of the crocodile (*Crocodylus*
*novaeguineae*). Comp. Biochem. Physiol. A.

[19] Wangensteen OD, Wilson D, Rahn H (1970). Diffusion of gases across the shell of the hen’s egg. Respir. Physiol..

[20] Ar A, Paganelli CV, Reeves RB, Greene DG, Rahn H (1974). The avian egg: Water vapor conductance, shell thickness, and functional pore area. Condor.

[21] Simkiss K (1986). Eggshell conductance—Fick’s or Stefan’s law?. Respir. Physiol..

[22] Deeming DC (2006). Ultrastructural and functional morphology of eggshells supports the idea that dinosaur eggs were incubated buried in a substrate. Palaeontology.

[23] Grellet-Tinner G, Fiorelli LE, Salvador RB (2012). Water vapor conductance of the Lower Cretaceous dinosaurian eggs from Sanagasta, La Rioja, Argentina: Paleobiological and paleoecological implications for South American faveoloolithid and megaloolithid eggs. Palaios.

[24] Tøien Ø, Paganelli CV, Rahn H, Johnson RR (1988). Diffusive resistance of avian eggshell pores. Respir. Physiol..

[25] Tøien Ø, Paganelli CV, Rahn H, Johnson RR (1987). Influence of eggshell pore shape on gas diffusion. J. Exp. Zool. Suppl..

[26] Tanaka K, Zelenitsky DK (2014). Comparisons between experimental and morphometric water vapor conductance in the eggs of extant birds and crocodiles: Implications for predicting nest type in dinosaurs. Can. J. Zool..

[27] Riley A, Sturrock CJ, Mooney SJ, Luck MR (2014). Quantification of eggshell microstructure using X-ray micro computed tomography. Br. Poult. Sci..

[28] Hechenleitner EM, Grellet-Tinner G, Foley M, Fiorelli LE, Thompson MB (2016). Micro-CT scan reveals an unexpected high-volume and interconnected pore network in a Cretaceous Sanagasta dinosaur eggshell. J. R. Soc. Interface.

[29] Willoughby B (2016). Micro-focus X-ray tomography study of the microstructure and morphometry of the eggshell of ostriches (*Struthio*
*camerus*). Anat. Rec..

[30] Vieco-Galvez D, Castro I, Morel PCH, Chua WH, Loh M (2021). The eggshell structure in *apteryx*; form, function, and adaptation. Ecol. Evol..

[31] Larriera, A. & Imhof, A. Proyecto yacaré. Cosecha de huevos para cría en granjas del género *Caiman* en la Argentina. in *Manejo de Fauna Silvestre en la Argentina. Programas de uso sustentable. Dirección de Fauna Silvestre, Secretaría de Ambiente y Desarrollo Sustentable, Buenos Aires* (eds. Bolkovic M.L. & Ramadori D.), 51–64 (2006).

[32] Montini JP, Piña CI, Larriera A, Siroski P, Verdade LM (2006). The relationship between nesting habitat and hatching success in *Caiman*
*latirostris* Crocodylia. Alligatoridae. Phyllomedusa.

[33] Sabath K (1991). Upper Cretaceous amniotic eggs from the Gobi Desert. Acta Palaeontol. Pol..

[34] Garcia G, Khosla A, Jafar SA, Sahni A, Vianey-Liaud M (2008). Eggshell microstructure and porosity of the Nicobar scrubfowl (*Megapodius*
*nicobarensis*, Great Nicobar Island, India). Palaeovertebrata.

[35] Grellet-Tinner G, Chiappe L, Norell M, Bottjer D (2006). Dinosaur eggs and nesting behaviors: A paleobiological investigation. Palaeogeogr. Palaeoclimatol. Palaeoecol..

[36] Hechenleitner EM, Grellet-Tinner G, Fiorelli LE (2015). What do giant titanosaur dinosaurs and modern Australasian megapodes have in common?. PeerJ.

[37] Norell MA (2020). The first dinosaur egg was soft. Nature.

[38] Grellet-Tinner G (2006). Phylogenetic interpretation of eggs and eggshells: Implications for phylogeny of Palaeognathae. Alcheringa.

[39] Donaire M, López-Martínez N (2009). Porosity of Late Paleocene *Ornitholithus* eggshells (Tremp Fm, south-central Pyrenees, Spain): Palaeoclimatic implications. Palaeogeogr. Palaeoclimatol. Palaeoecol..

[40] Grellet-Tinner G, Lindsay S, Thompson MB (2017). The biomechanical, chemical and physiological adaptations of the eggs of two Australian megapodes to their nesting strategies and their implications for extinct titanosaur dinosaurs. J. Microsc..

[41] Lawver DR, Boyd CA (2018). An avian eggshell from the Brule Formation (Oligocene) of North Dakota. J. Vertebr. Paleontol..

[42] Norell MA (1994). A theropod dinosaur embryo and the affinities of the Flaming Cliffs dinosaur eggs. Science.

[43] Zelenitsky DK, Carpenter K, Currie PJ (2000). First record of elongatoolithid theropod eggshell from North America: The Asian oogenus *Macroelongatoolithus* from the Lower Cretaceous of Utah. J. Vertebr. Paleontol..

[44] Grellet-Tinner G, Chiappe LM, Coria RA (2004). Eggs of titanosaurid sauropods from the Upper Cretaceous of Auca Mahuevo (Argentina). Can. J. Earth Sci..

[45] Grellet-Tinner G, Makovicky P (2006). A possible egg of the dromaeosaur *Deinonychus*
*antirrhopus*: Phylogenetic and biological implications. Can. J. Earth Sci..

[46] Araújo R (2013). Filling the gaps of dinosaur eggshell phylogeny: Late Jurassic Theropod clutch with embryos from Portugal. Sci. Rep..

[47] Vila B, Sellés AG, Beetschen JC (2017). The controversial Les Labadous eggshells: A new and peculiar dromaeosaurid (Dinosauria: Theropoda) ootype from the Upper Cretaceous of Europe. Cretac. Res..

[48] Choi, S., Moreno‐Azanza, M., Csiki‐Sava, Z., Prondvai, E. & Lee, Y. Comparative crystallography suggests maniraptoran theropod affinities for latest Cretaceous European ‘geckoid’ eggshell. *Pap. Palaeontol.* spp2.1294 (2020).

[49] de Marsola JC, Batezelli A, Montefeltro FC, Grellet-Tinner G, Langer MC (2016). Palaeoenvironmental characterization of a crocodilian nesting site from the Late Cretaceous of Brazil and the evolution of crocodyliform nesting strategies. Palaeogeogr. Palaeoclimatol. Palaeoecol..

[50] Fernandez Blanco, M. V. Análisis morfológico del esqueleto de las especies argentinas del género Caiman (Alligatoridae: Caimaninae). Aportes al conocimiento de la historia evolutiva de los alligatóridos sudamericanos. (Universidad Nacional de La Plata, 2018).

[51] Fernandez Blanco, M. V. & Bona, P. Embryonic development of cranial bones in two Argentinian caiman species (*Caiman latirostris* and *Caiman yacare*) with new insights into the homology of some controversial elements. *in prep*.

[52] Corona TB, Warburton SJ (2000). Regional hypoxia elicits regional changes in chorioallantoic membrane vascular density in alligator but not chicken embryos. Comp. Biochem. Physiol. A.

[53] Board RG, Tullett SG, Perrott HR (1977). An arbitrary classification of the pore systems in avian eggs. J. Zool..

[54] Simkiss K (1961). Calcium metabolism and avian reproduction. Biol. Rev..

[55] Ar A, Rahn H (1985). Pores in avian eggshells: Gas conductance, gas exchange and embryonic growth rate. Respir. Physiol..

[56] Wink CS, Elsey RM (1994). Morphology of shells from viable and nonviable eggs of the Chinese alligator (*Alligator*
*sinensis*). J. Morphol..

[57] Tyler C, Simkiss K (1959). A study of the egg shells of ratite birds. Proc. Zool. Soc. London.

[58] Board RG, Tullett SG (1975). The pore arrangement in the emu (*Dromaius*
*novaehollandiae*) eggshell as shown by plastic models. J. Microsc..

[59] Board RG (1982). Properties of avian egg shells and their adaptive value. Biol. Rev..

[60] Board RG, Perrott HR, Love G, Seymour RS (1982). A novel pore system in the eggshells of the mallee fowl, *Leipoa*
*ocellata*. J. Exp. Zool..

[61] Williams DLG, Seymour RS, Kerourio P (1984). Structure of fossil dinosaur eggshell from the Aix Basin, France. Palaeogeogr. Palaeoclimatol. Palaeoecol..

[62] Bravo AM, Vila B, Galobart À, Oms O (2005). Restos de huevos de dinosaurio en el Cretácico Superior del sinclinal de Vallcebre (Berguedà, provincia de Barcelona). Rev. Española Paleontol..

[63] Rasskin-Gutman D, Elez J, Esteve-Altava B, López-Martínez N (2013). Reconstruction of the internal structure of the pore system of a complex dinosaur eggshell (*Megaloolithus*
*siruguei*). Spanish J. Palaeontol..

[64] Larriera, A. Áreas de nidificación y momento óptimo de cosecha de huevos de *Caiman latirostris* en Santa Fe, Argentina. in *La conservación y el manejo de caimanes y cocodrilos de América Latina 1. Fundación Banco Bica, Santo Tomé* (eds. Larriera, A. & Verdade, L. M.) 221–232 (1995).

[65] Iungman J, Piña CI, Siroski P (2008). Embryological development of *Caiman*
*latirostris* (Crocodylia: Alligatoridae). Genesis.

[66] R Core Team. *R: A language and environment for statistical computing* (2022).

[67] Wickham H (2016). ggplot2: Elegant Graphics for Data Analysis.

