# Supplementary material for: Unexpected morphological variability in the eggshells of the South American caimans Caiman latirostris and Caiman yacare
Source: Sci Rep. 2023 Mar 25;13:4894. doi: 10.1038/s41598-023-31837-9 (PMC10039913; doi:10.1038/s41598-023-31837-9)
Supplement: Supplementary file 1 — Supplementary Information 1. [file 41598_2023_31837_MOESM1_ESM.pdf]

Supplementary Information

for

Unexpected morphological variability in the eggshells of the South American caimans *Caiman latirostris* and *Caiman yacare*

by

E. Martín Hechenleitner*, María V. Fernandez Blanco*, Segundo R. Núñez-Campero, Lucas E. Fiorelli, and Paula Bona

*emails: emhechenleitner@gmail.com; martinh@conicet.gov.ar (EMH); victoriafernandezblanco@yahoo.com.ar (MVFB)

This file includes the following supplementary methods and results:

Micro-CT samples	2
Segmentation of micro-CT scans	2
Micro-CT rendering	3
Eggshell measurements	27
Pore cross-sectional area and water vapor conductance (G_{H2O})	28

Micro-CT samples

For the present study, we scanned six eggshell fragments for *Caiman latirostris* and six for *Caiman yacare*. Each sample corresponds to a different developmental stage. The collection numbers are given in the Supplementary Table S1.

Eggshells MLP.R.6800	Acronyms used in the main text	Embryonic stage
**MLP.R.6800-1**	CL1	15
**MLP.R.6800-6**	CL2	17/18
**MLP.R.6800-10**	CL3	18
**MLP.R.6800-12**	CL4	19
**MLP.R.6800-17**	CL5	20
**MLP.R.6800-29**	CL6	21
**MLP.R.6800-36**	CY1	17/18
**MLP.R.6800-39**	CY2	18
**MLP.R.6800-48**	CY3	19
**MLP.R.6800-53**	CY4	20
**MLP.R.6800-60**	CY5	22
**MLP.R.6800-66**	CY6	23

Supplementary Table S1. Eggshell samples of *C. latirostris* (CL) and C. yacare (CY).

Segmentation of micro-CT scans

After reorienting the sections of each eggshell such that their surface is approximately "parallel" to the z-axis. We manually segmented the pore canals that traverse the shell (Supplementary Fig. S1).

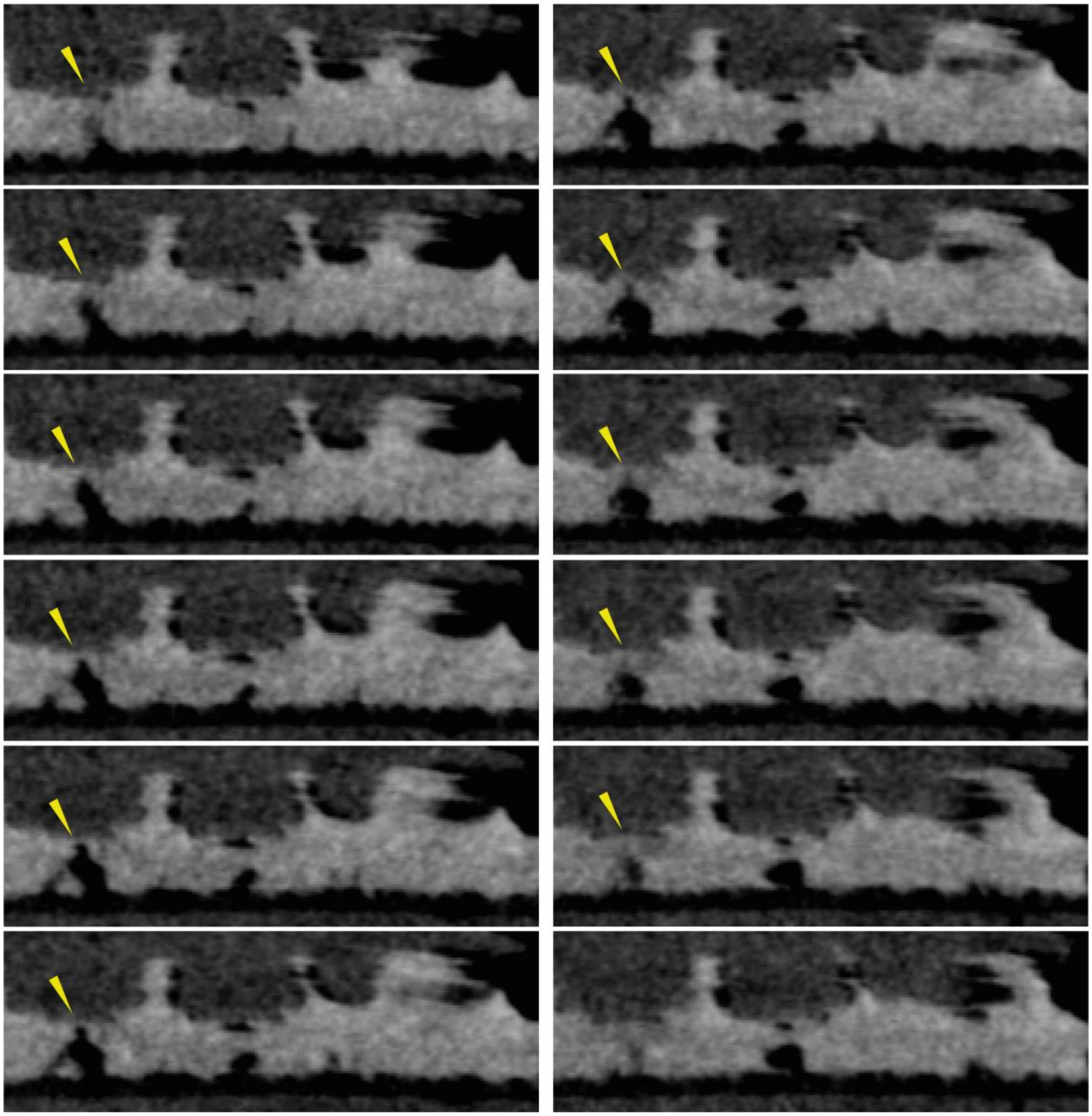

Supplementary Figure S1. Tangential sections of the eggshell sample CL6. Low intensity pixels (black) correspond to empty spaces. When these spaces connect both surfaces, they are interpreted as a pore canal. The yellow arrow points a pore canal that shows a single external aperture (top) and two independent inner apertures (bottom). Part of an amphora-shaped pore is also visible at the center of most slices.

Micro-CT rendering

The following figures include screenshots of all the surface views of the eggshells and pore canals reconstructed for *C. latirostris* (Supplementary Fig. 1-12, and Supplementary Renderings S1) and *C. yacare* (Supplementary Fig. 13-25, and Supplementary Renderings

S2). Each eggshell sample was divided in six sectors (A-F) of about 9 mm^2 (see Methods). Measurements of the pore cross-sectional areas at each sector of each eggshell fragment of *C. latirostris* and *C. yacare* are listed in Supplementary Tables S3 and S4, respectively.

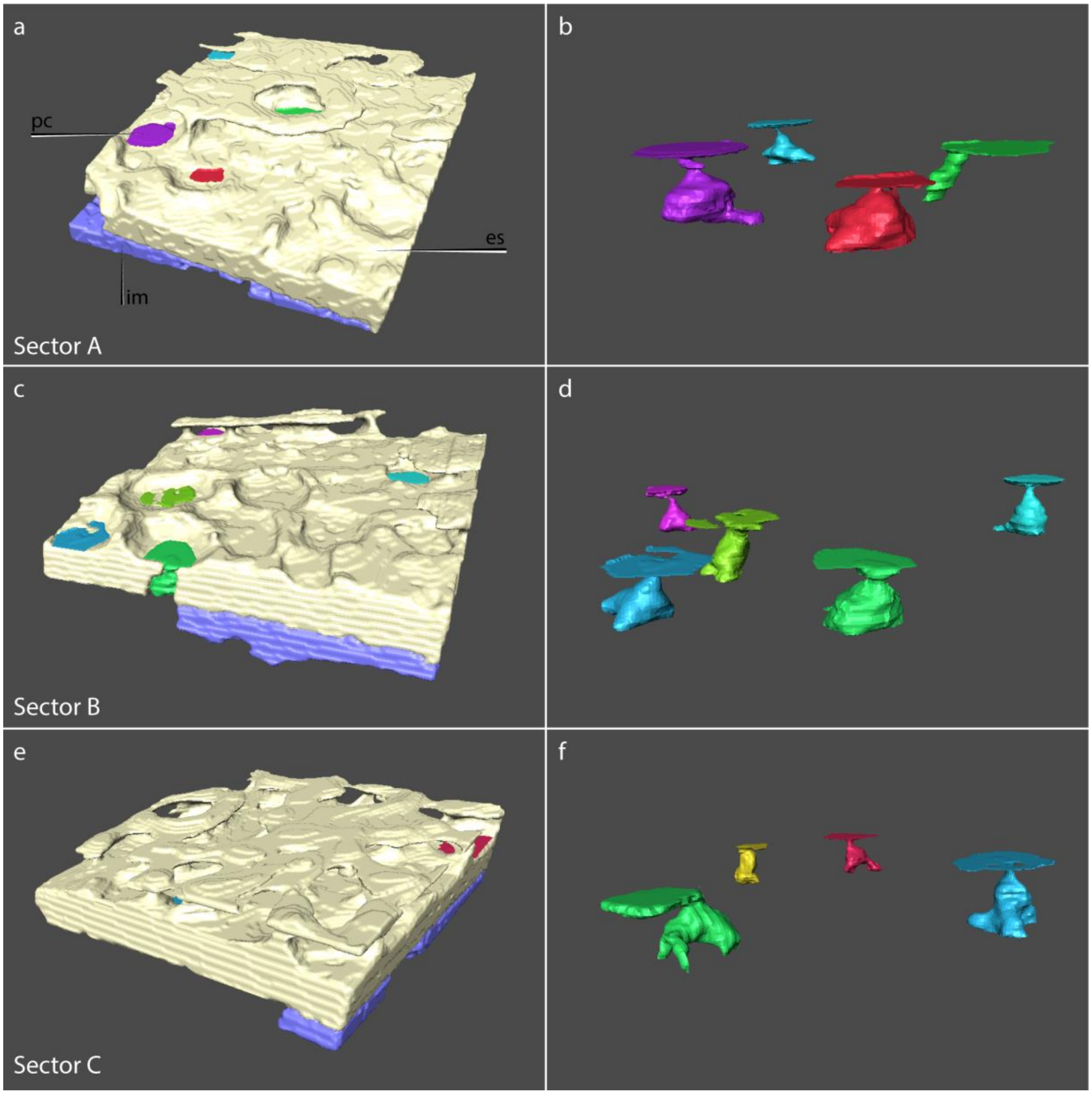

Supplementary Figure S2. Eggshell CL1. (a, b) Sector A. (c, d) Sector B. (e, f) Sector C. Note that some pore canals have extremely wide external ends. As the pore canals get thinner outwards, we included a few outer slices to be sure that all the pore slices were recorded. Abbreviations: es, eggshell; im, inner shell membrane; pc, pore canal. Each sector measures ~9 mm^2.

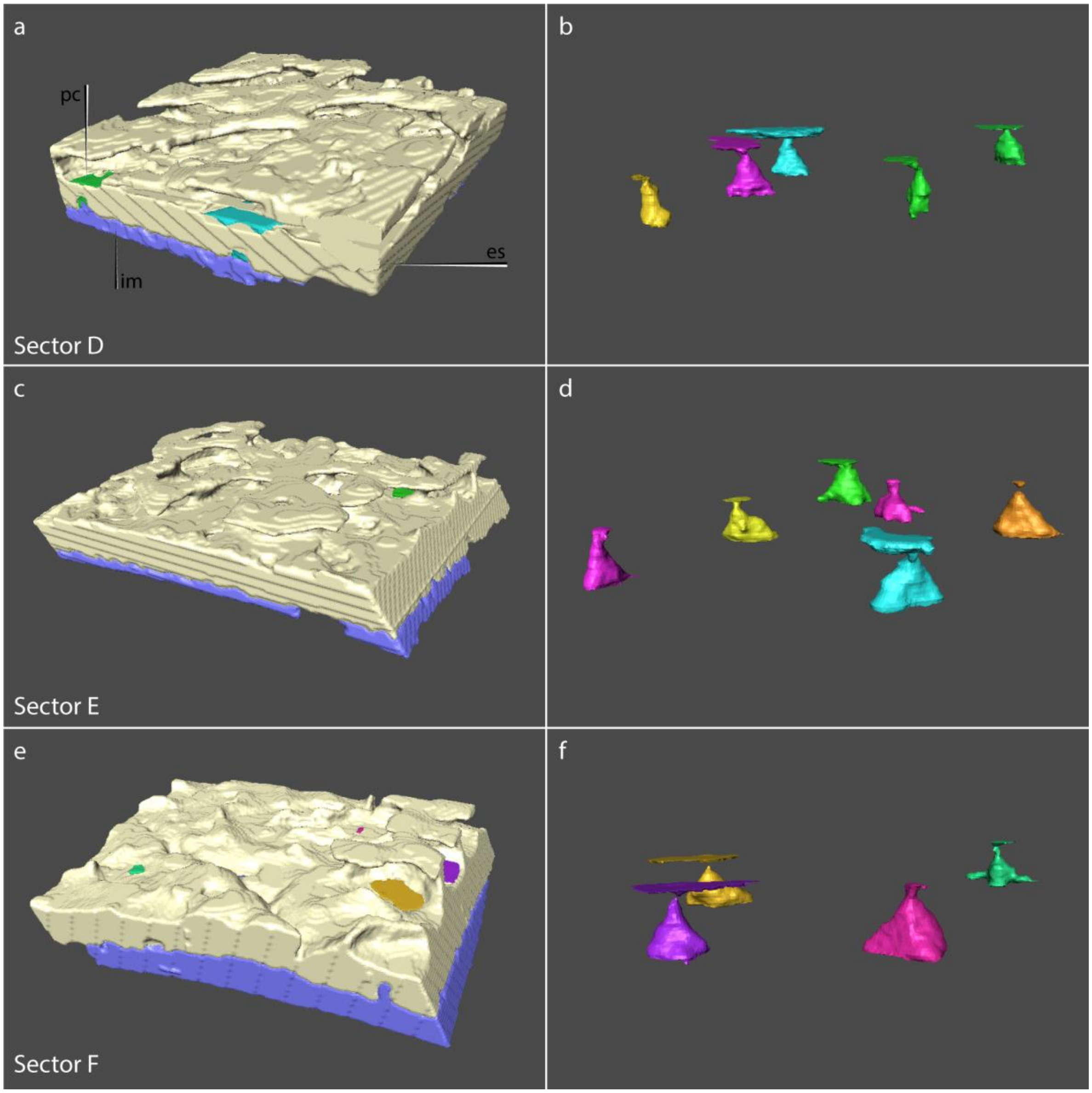

Supplementary Figure S3. Eggshell CL1. (a, b) Sector D. (c, d) Sector E. (e, f) Sector F. Note that some pore canals have extremely wide external ends. As the pore canals get thinner outwards, we included a few outer slices to be sure that all the pore slices were recorded. Abbreviations: es, eggshell; im, inner shell membrane; pc, pore canal. Each sector measures ~9 mm^2.

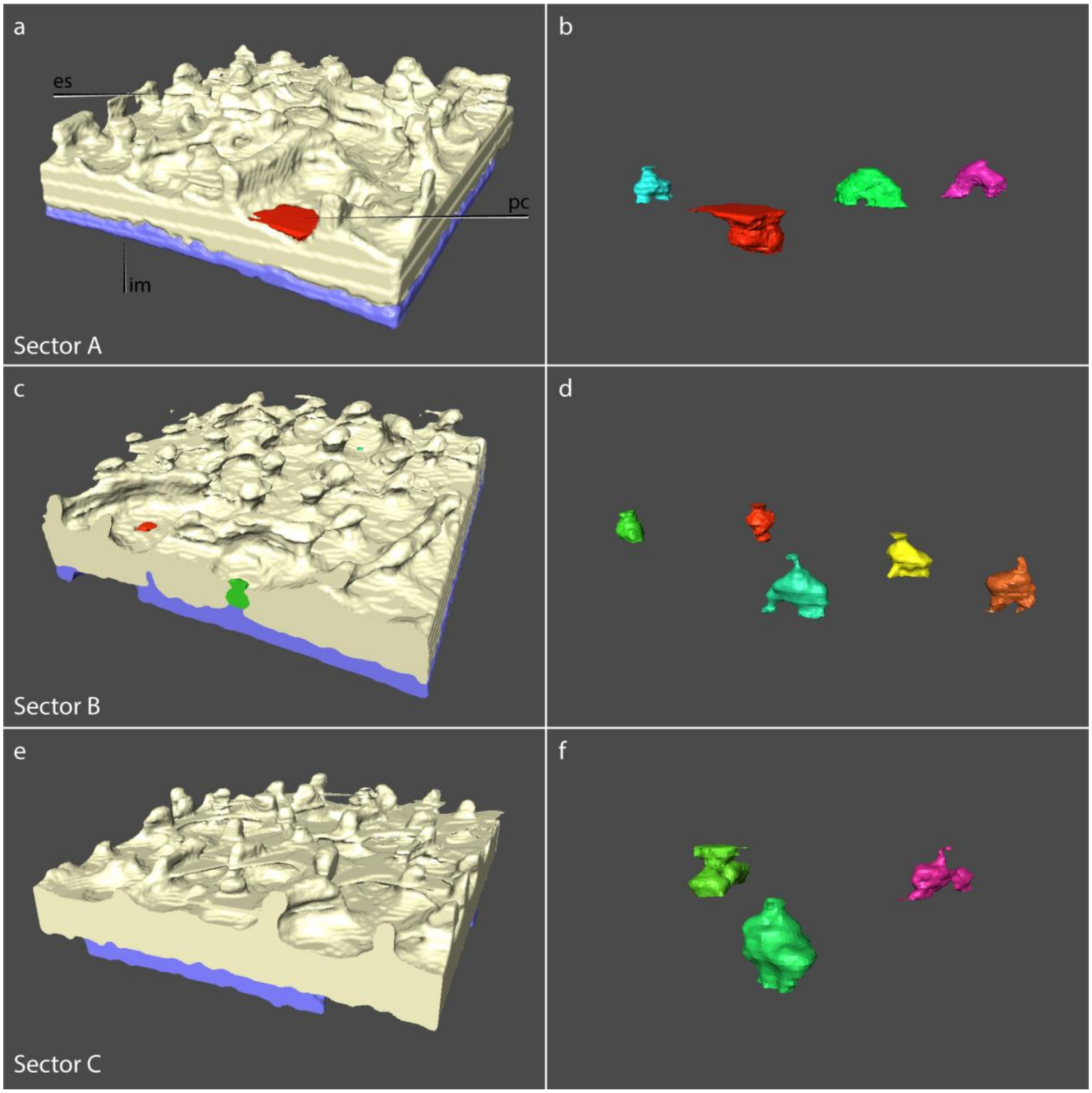

Supplementary Figure S4. Eggshell CL2. (a, b) Sector A. (c, d) Sector B. (e, f) Sector C. Note that some pore canals have extremely wide external ends. As the pore canals get thinner outwards, we included a few outer slices to be sure that all the pore slices were recorded. Abbreviations: es, eggshell; im, inner shell membrane; pc, pore canal. Each sector measures ~9 mm^2.

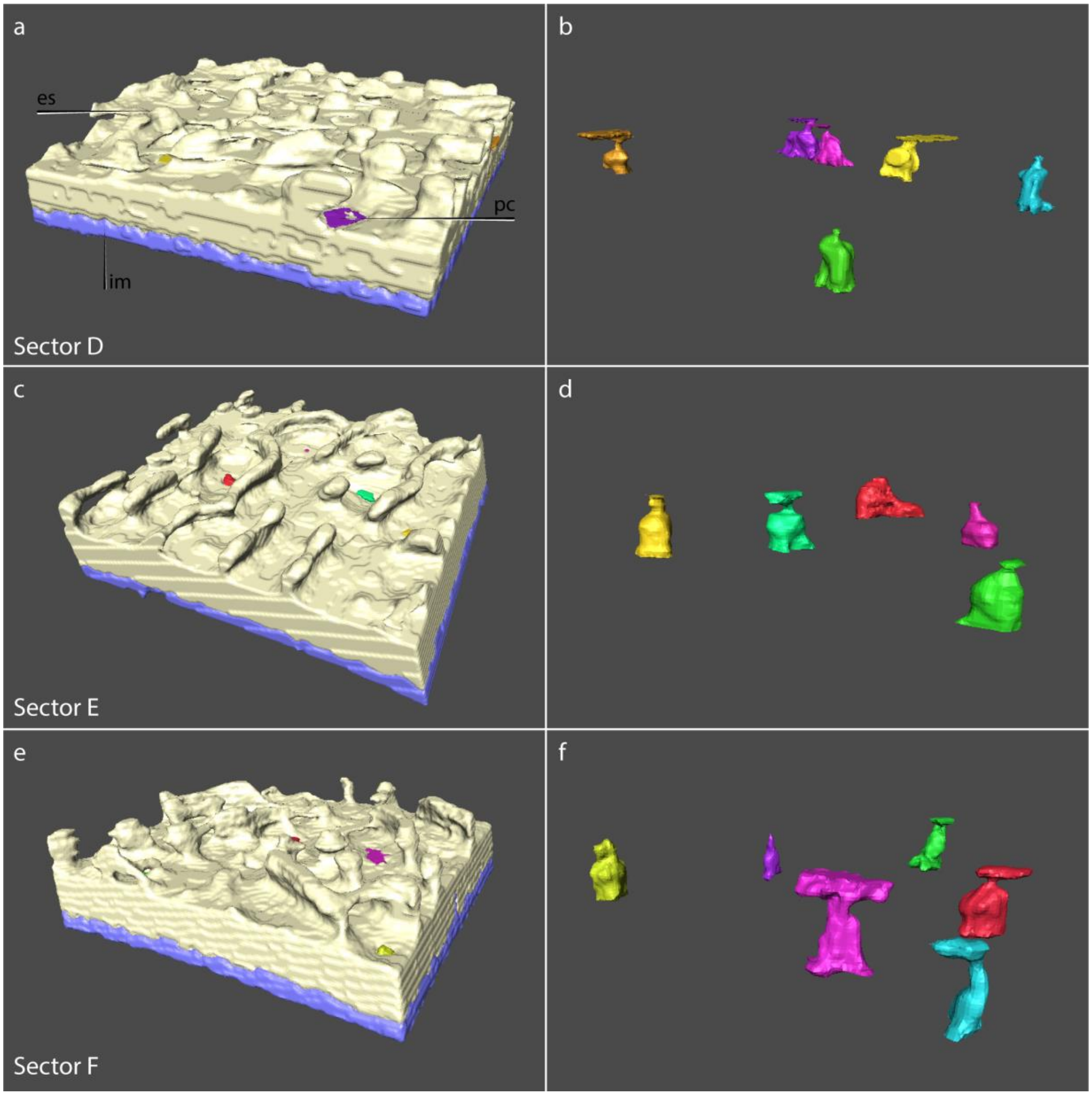

Supplementary Figure S5. Eggshell CL2. (a, b) Sector D. (c, d) Sector E. (e, f) Sector F. Note that some pore canals have extremely wide external ends. As the pore canals get thinner outwards, we included a few outer slices to be sure that all the pore slices were recorded. Abbreviations: es, eggshell; im, inner shell membrane; pc, pore canal. Each sector measures ~9 mm^2.

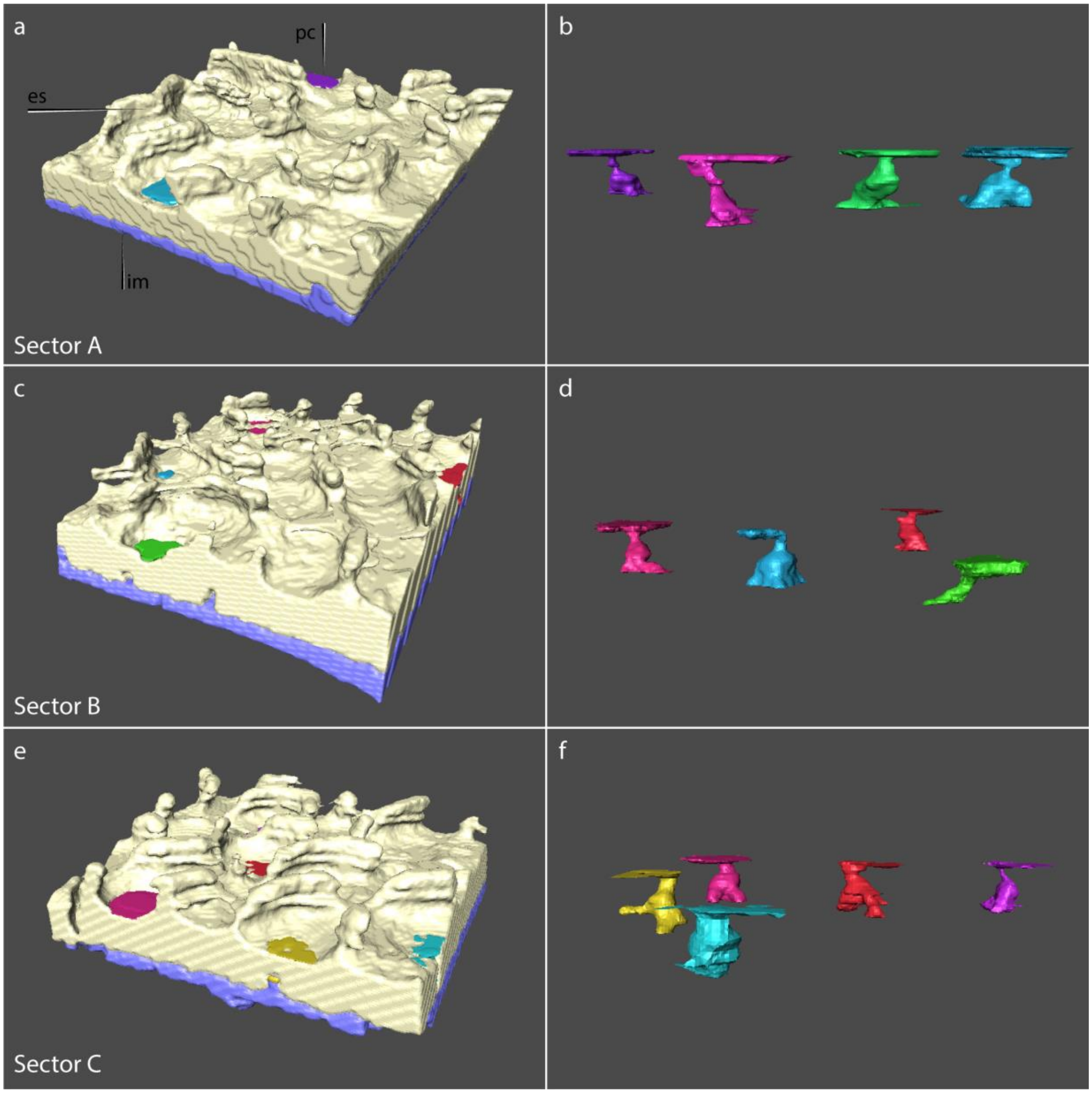

Supplementary Figure S6. Eggshell CL3. (a, b) Sector A. (c, d) Sector B. (e, f) Sector C. Note that some pore canals have extremely wide external ends. As the pore canals get thinner outwards, we included a few outer slices to be sure that all the pore slices were recorded. Abbreviations: es, eggshell; im, inner shell membrane; pc, pore canal. Each sector measures ~9 mm^2.

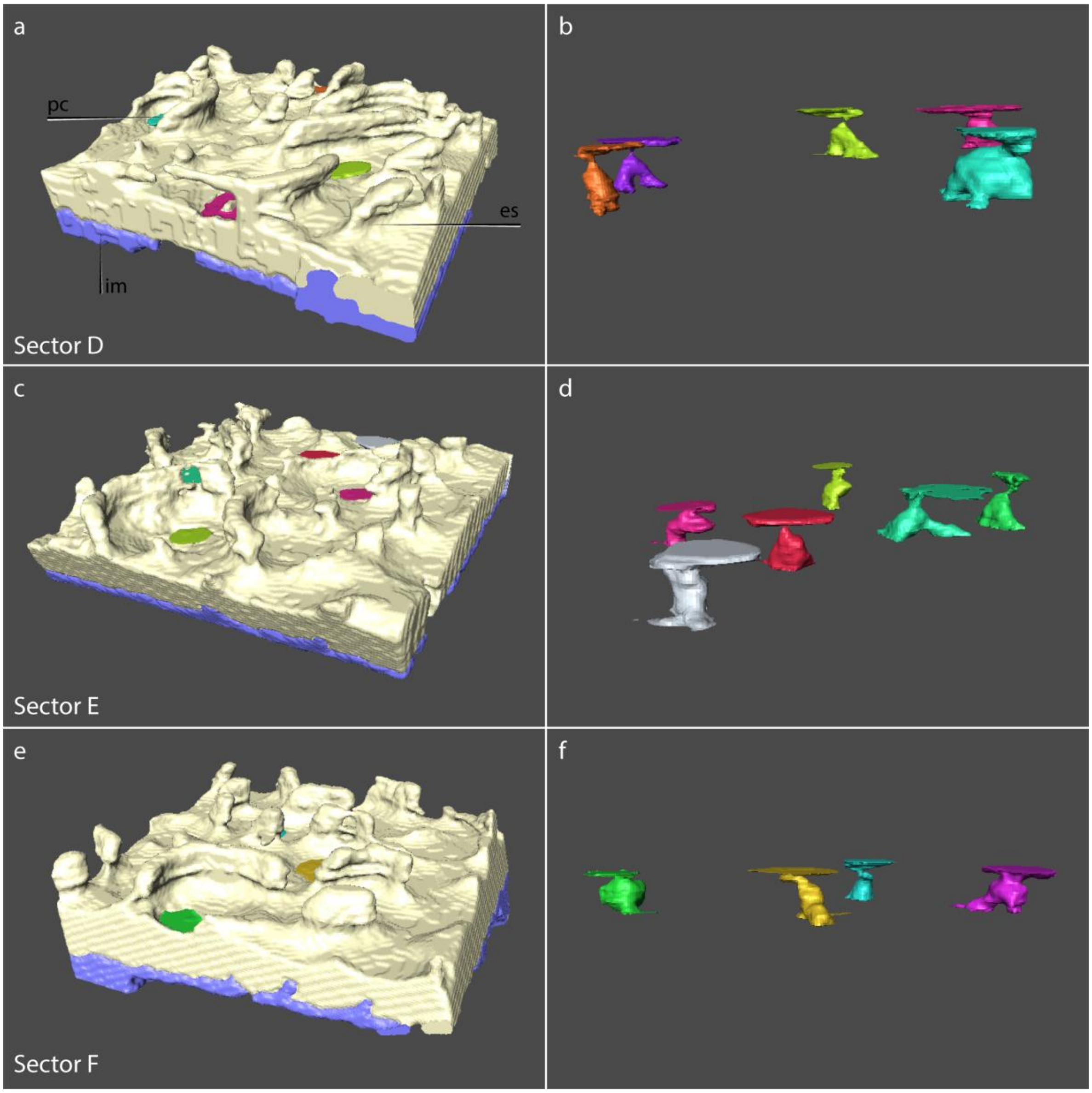

Supplementary Figure S7. Eggshell CL3. (a, b) Sector D. (c, d) Sector E. (e, f) Sector F. Note that some pore canals have extremely wide external ends. As the pore canals get thinner outwards, we included a few outer slices to be sure that all the pore slices were recorded. Abbreviations: es, eggshell; im, inner shell membrane; pc, pore canal. Each sector measures ~9 mm^2.

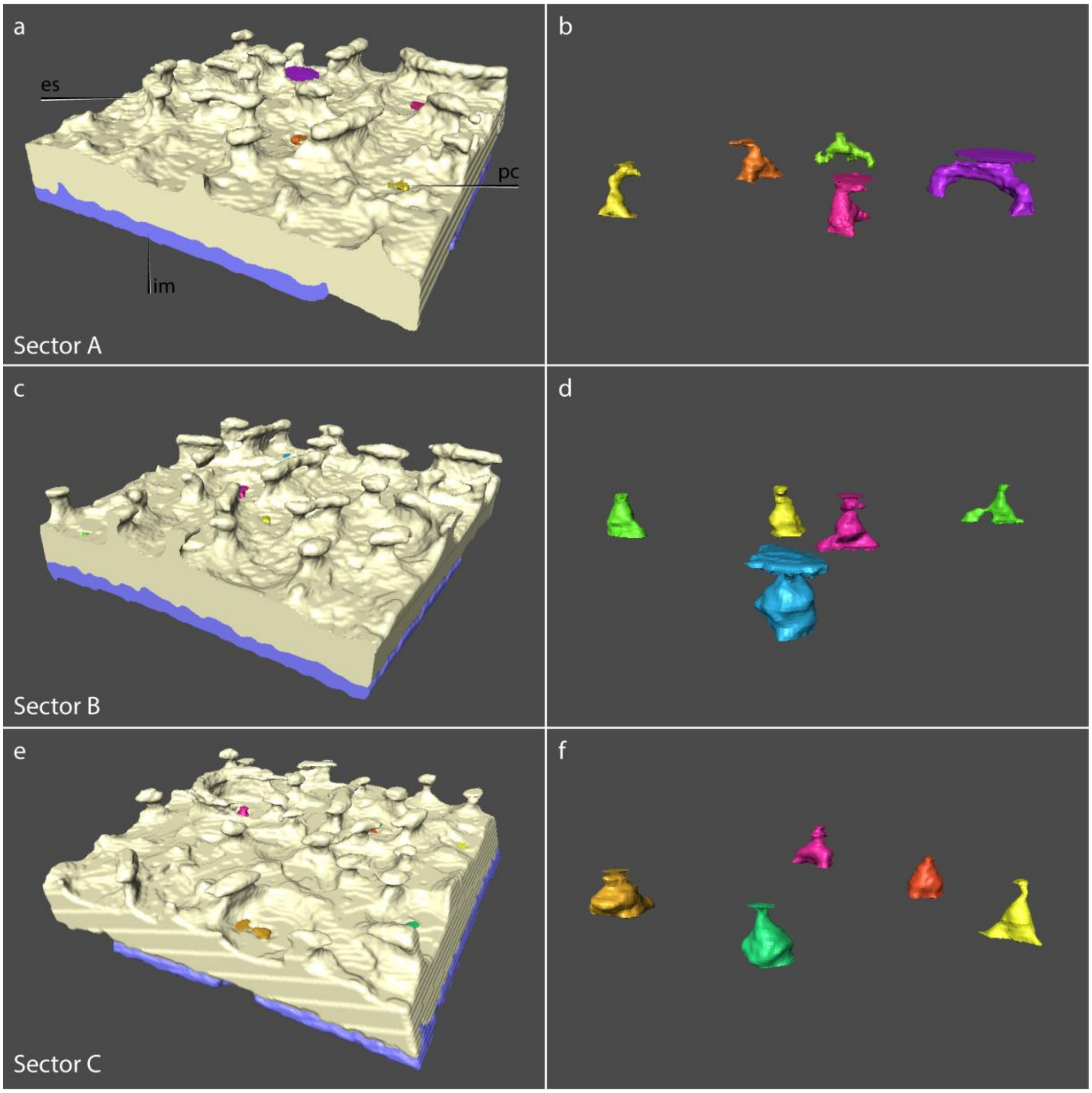

Supplementary Figure S8. Eggshell CL4. (a, b) Sector A. (c, d) Sector B. (e, f) Sector C. Note that some pore canals have extremely wide external ends. As the pore canals get thinner outwards, we included a few outer slices to be sure that all the pore slices were recorded. Abbreviations: es, eggshell; im, inner shell membrane; pc, pore canal. Each sector measures ~9 mm^2.

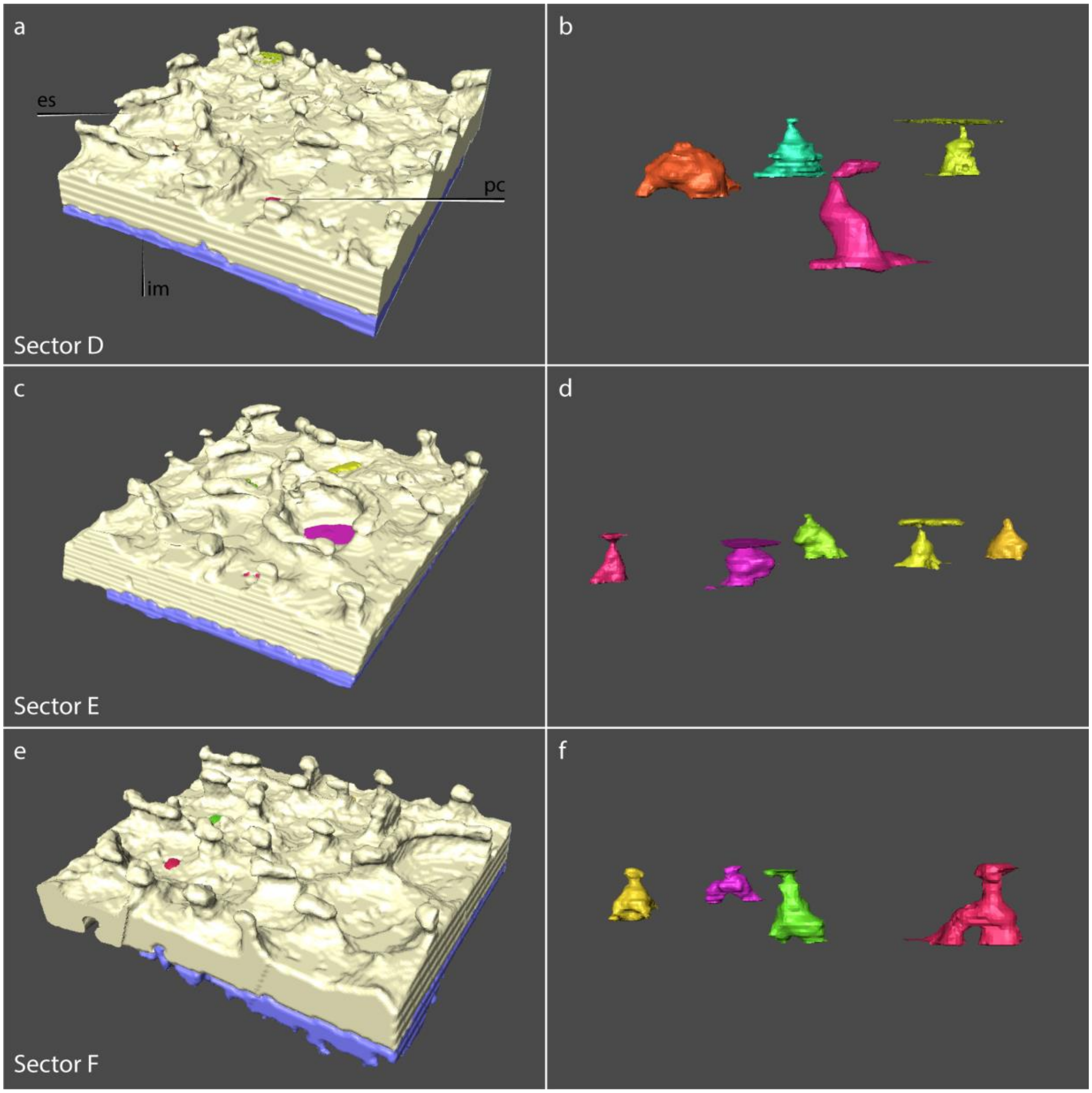

Supplementary Figure S9. Eggshell CL4. (a, b) Sector D. (c, d) Sector E. (e, f) Sector F. Note that some pore canals have extremely wide external ends. As the pore canals get thinner outwards, we included a few outer slices to be sure that all the pore slices were recorded. Abbreviations: es, eggshell; im, inner shell membrane; pc, pore canal. Each sector measures ~9 mm^2.

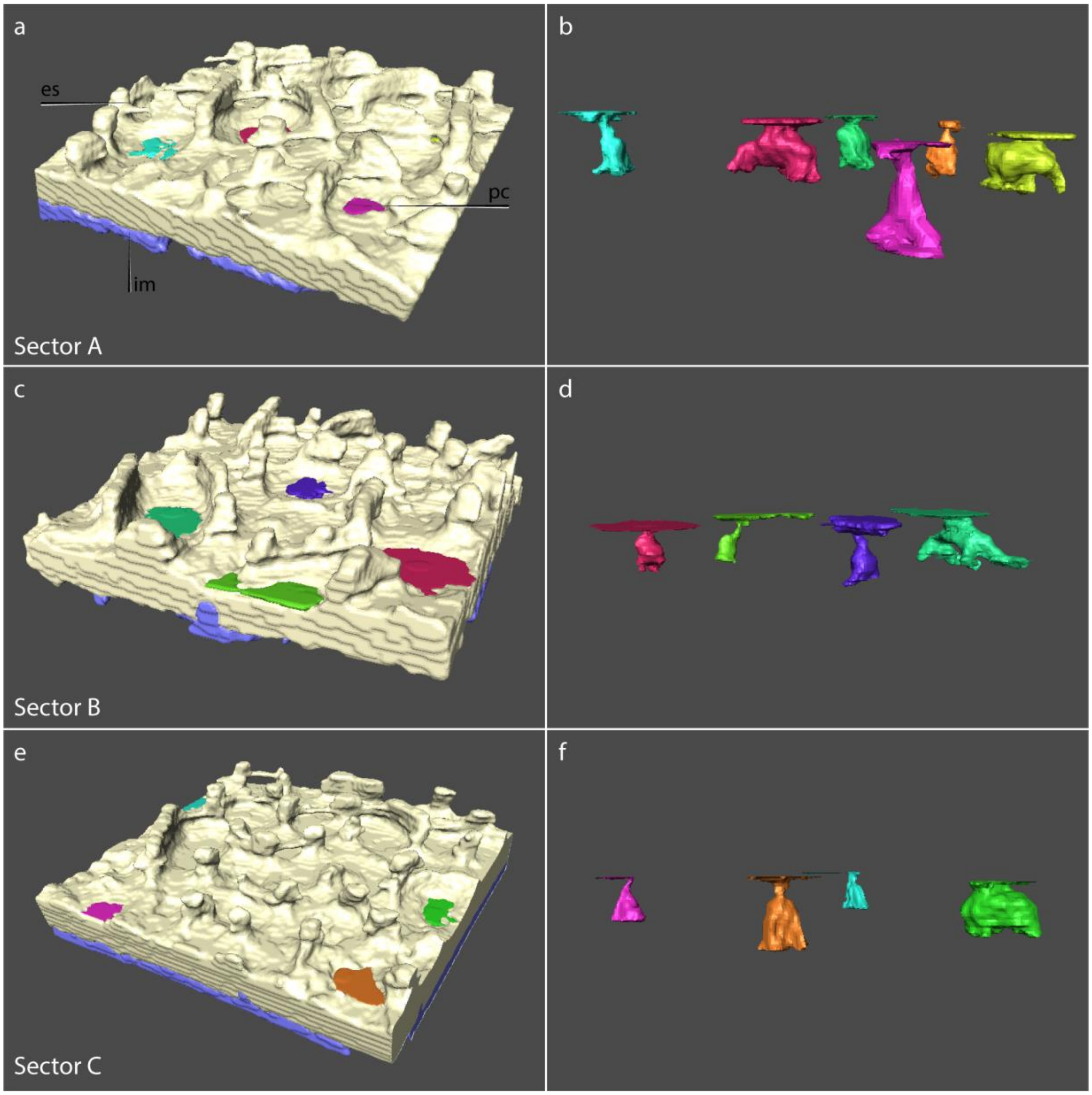

Supplementary Figure S10. Eggshell CL5. (a, b) Sector A. (c, d) Sector B. (e, f) Sector C. Note that some pore canals have extremely wide external ends. As the pore canals get thinner outwards, we included a few outer slices to be sure that all the pore slices were recorded. Abbreviations: es, eggshell; im, inner shell membrane; pc, pore canal. Each sector measures ~9 mm^2.

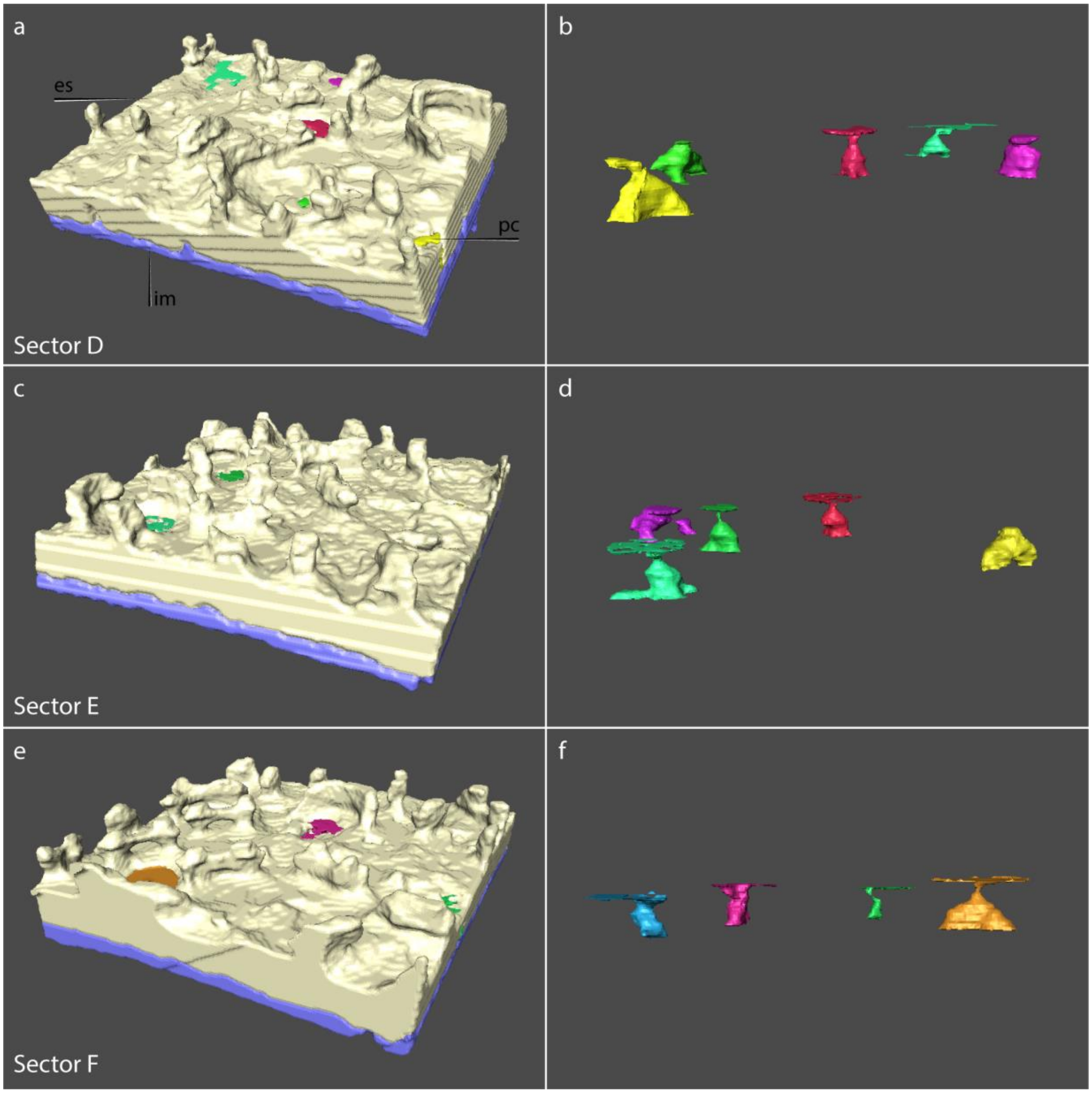

Supplementary Figure S11. Eggshell CL5. (a, b) Sector D. (c, d) Sector E. (e, f) Sector F. Note that some pore canals have extremely wide external ends. As the pore canals get thinner outwards, we included a few outer slices to be sure that all the pore slices were recorded. Abbreviations: es, eggshell; im, inner shell membrane; pc, pore canal. Each sector measures ~9 mm^2.

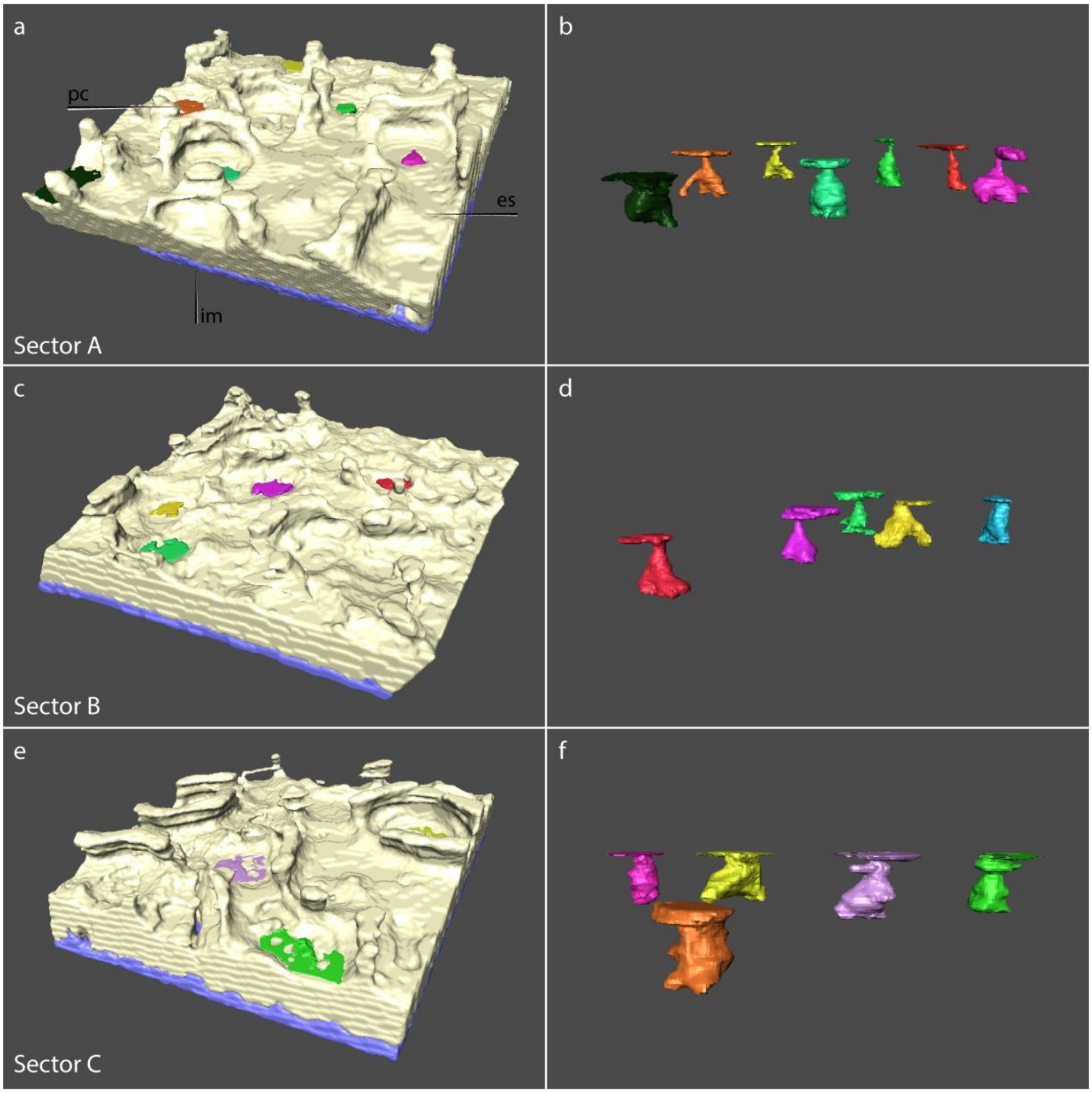

Supplementary Figure S12. Eggshell CL6. (a, b) Sector A. (c, d) Sector B. (e, f) Sector C. Note that some pore canals have extremely wide external ends. As the pore canals get thinner outwards, we included a few outer slices to be sure that all the pore slices were recorded. Abbreviations: es, eggshell; im, inner shell membrane; pc, pore canal. Each sector measures ~9 mm^2.

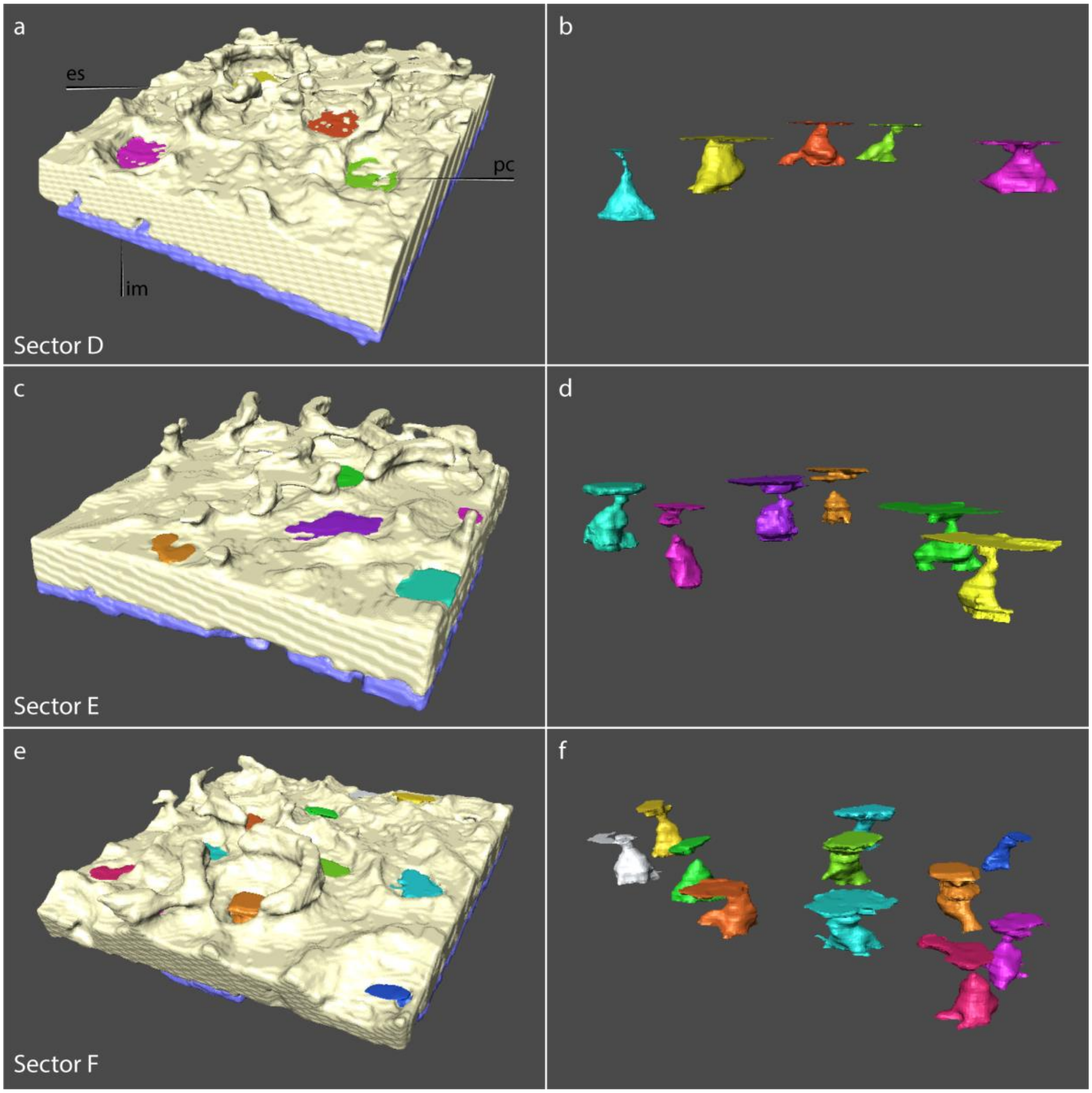

Supplementary Figure S13. Eggshell CL6. (a, b) Sector D. (c, d) Sector E. (e, f) Sector F. Note that some pore canals have extremely wide external ends. As the pore canals get thinner outwards, we included a few outer slices to be sure that all the pore slices were recorded. Abbreviations: es, eggshell; im, inner shell membrane; pc, pore canal. Each sector measures ~9 mm^2.

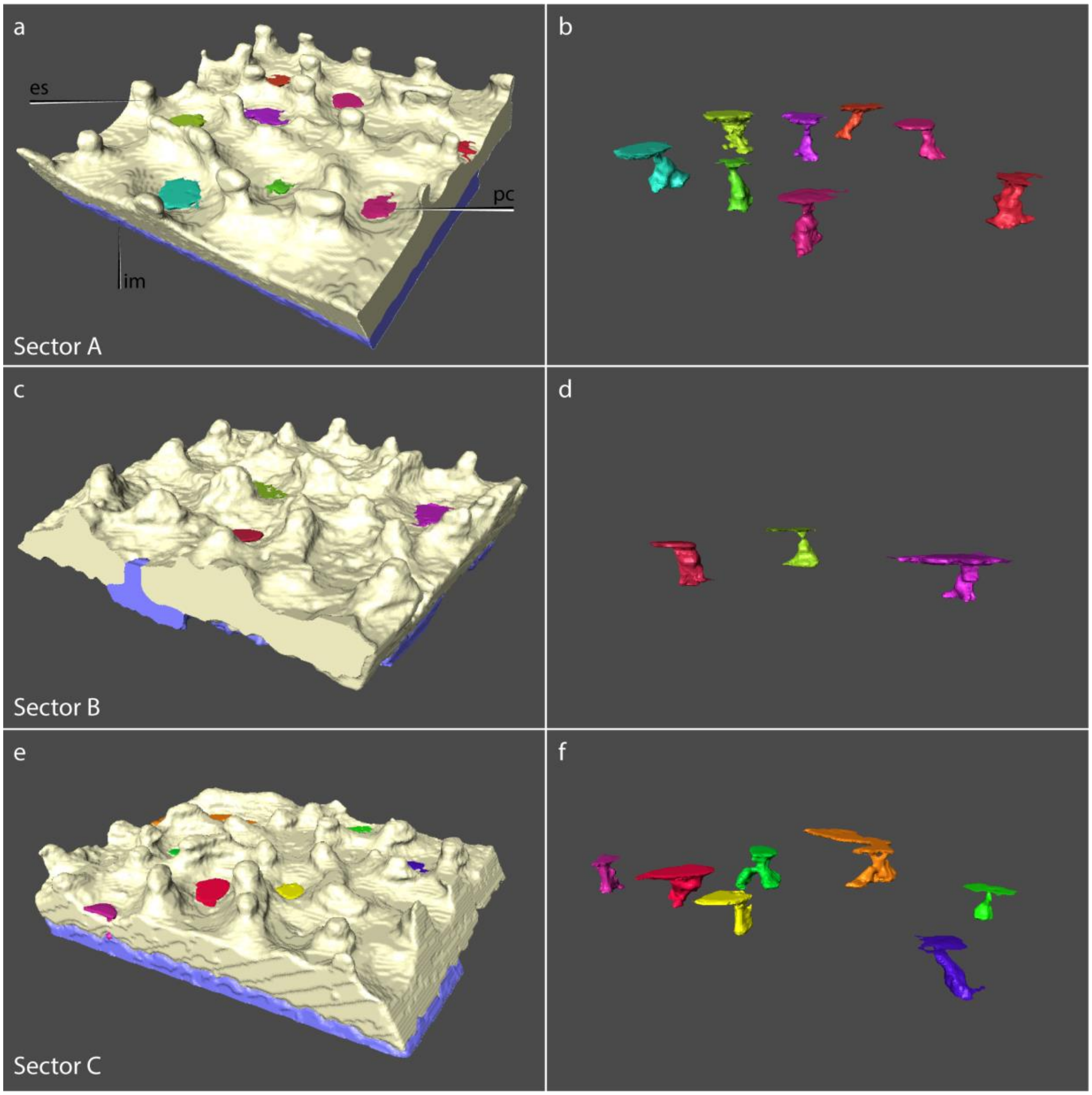

Supplementary Figure S14. Eggshell CY1. (a, b) Sector A. (c, d) Sector B. (e, f) Sector C. Note that some pore canals have extremely wide external ends. As the pore canals get thinner outwards, we included a few outer slices to be sure that all the pore slices were recorded. Abbreviations: es, eggshell; im, inner shell membrane; pc, pore canal. Each sector measures ~9 mm^2.

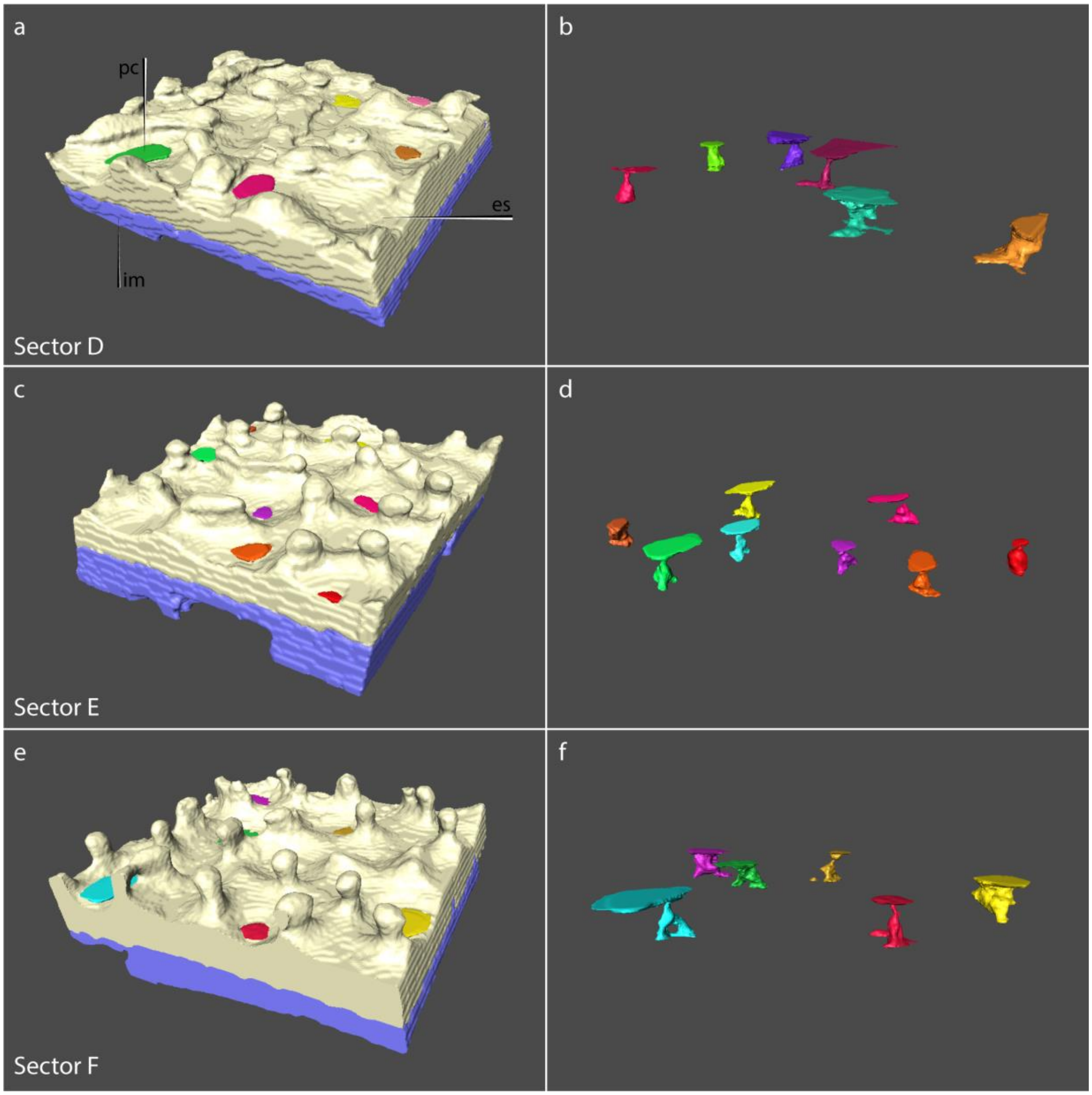

Supplementary Figure S15. Eggshell CY1. (a, b) Sector D. (c, d) Sector E. (e, f) Sector F. Note that some pore canals have extremely wide external ends. As the pore canals get thinner outwards, we included a few outer slices to be sure that all the pore slices were recorded. Abbreviations: es, eggshell; im, inner shell membrane; pc, pore canal. Each sector measures ~9 mm^2.

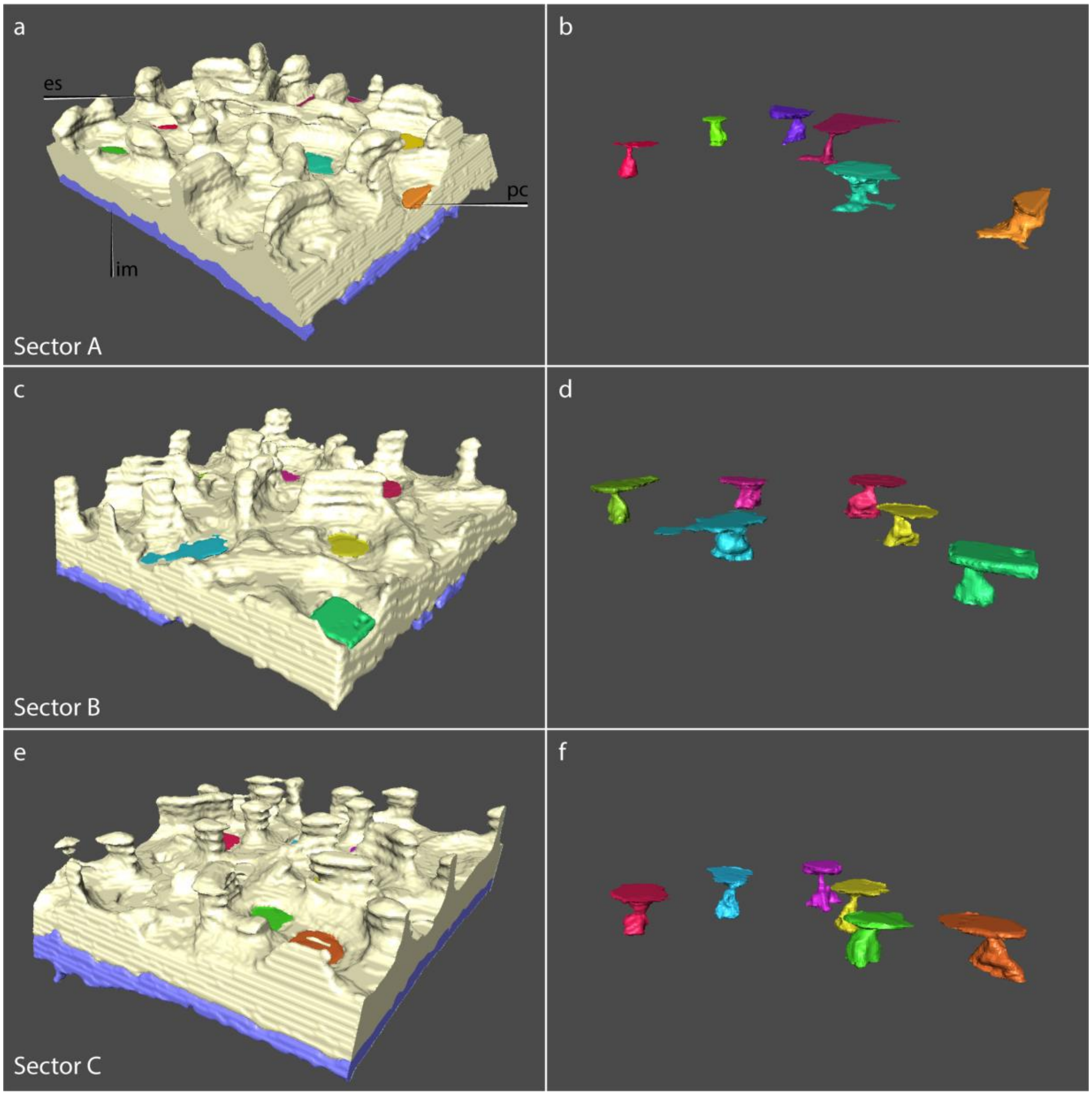

Supplementary Figure S16. Eggshell CY2. (a, b) Sector A. (c, d) Sector B. (e, f) Sector C. Note that some pore canals have extremely wide external ends. As the pore canals get thinner outwards, we included a few outer slices to be sure that all the pore slices were recorded. Abbreviations: es, eggshell; im, inner shell membrane; pc, pore canal. Each sector measures ~9 mm^2.

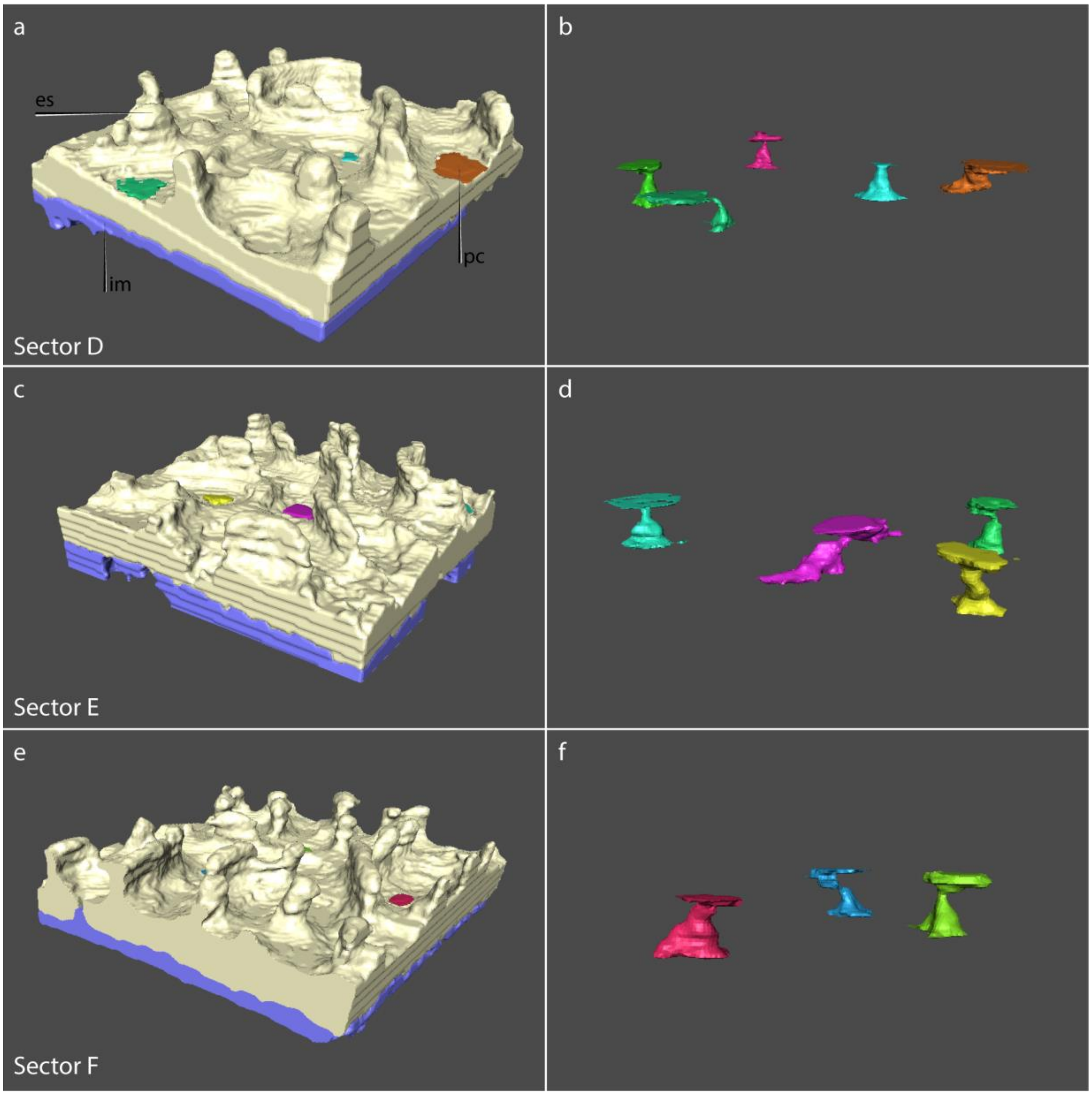

Supplementary Figure S17. Eggshell CY2. (a, b) Sector D. (c, d) Sector E. (e, f) Sector F. Note that some pore canals have extremely wide external ends. As the pore canals get thinner outwards, we included a few outer slices to be sure that all the pore slices were recorded. Abbreviations: es, eggshell; im, inner shell membrane; pc, pore canal. Each sector measures ~9 mm^2.

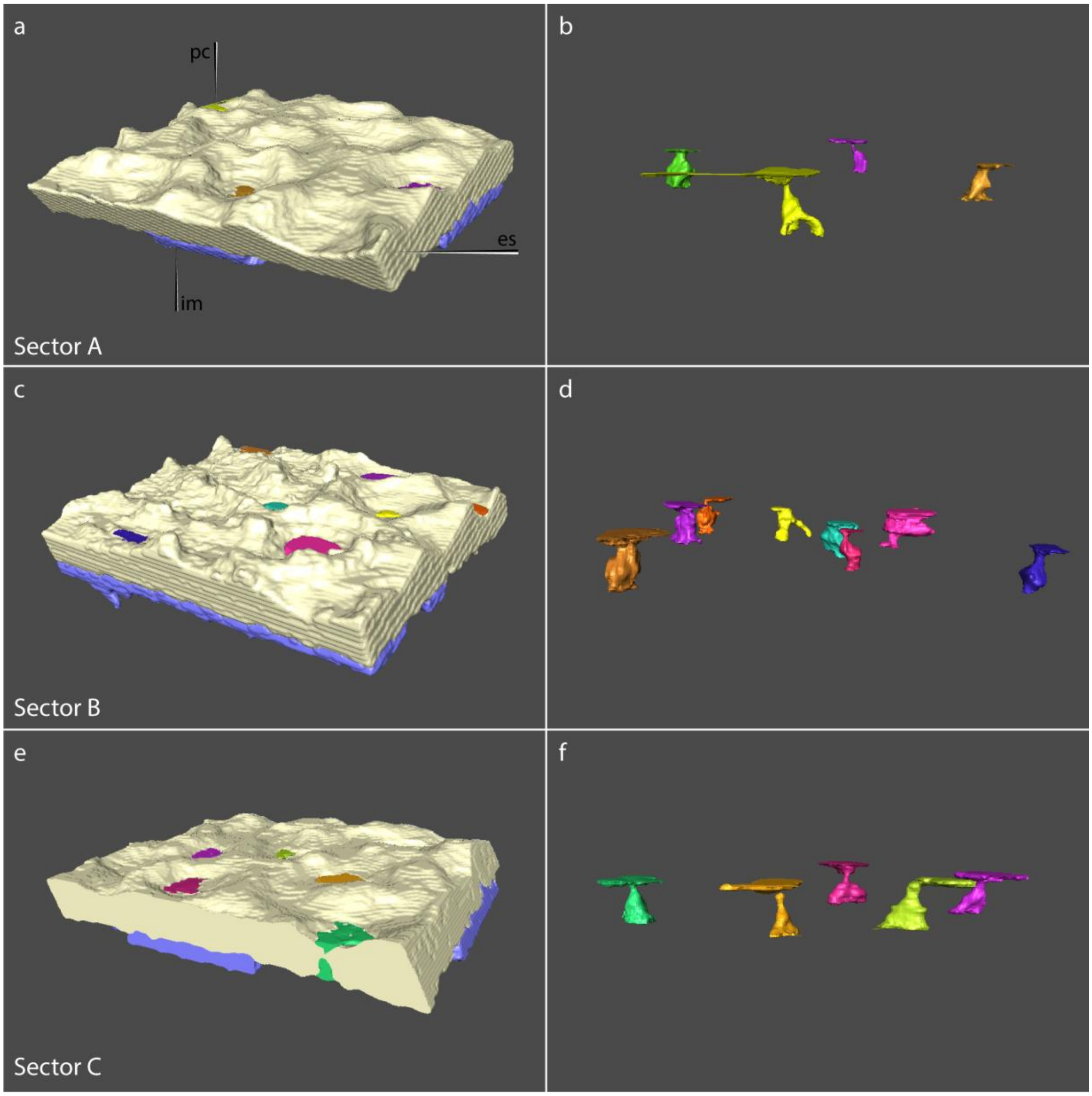

Supplementary Figure S18. Eggshell CY3. (a, b) Sector A. (c, d) Sector B. (e, f) Sector C. Note that some pore canals have extremely wide external ends. As the pore canals get thinner outwards, we included a few outer slices to be sure that all the pore slices were recorded. Abbreviations: es, eggshell; im, inner shell membrane; pc, pore canal. Each sector measures ~9 mm^2.

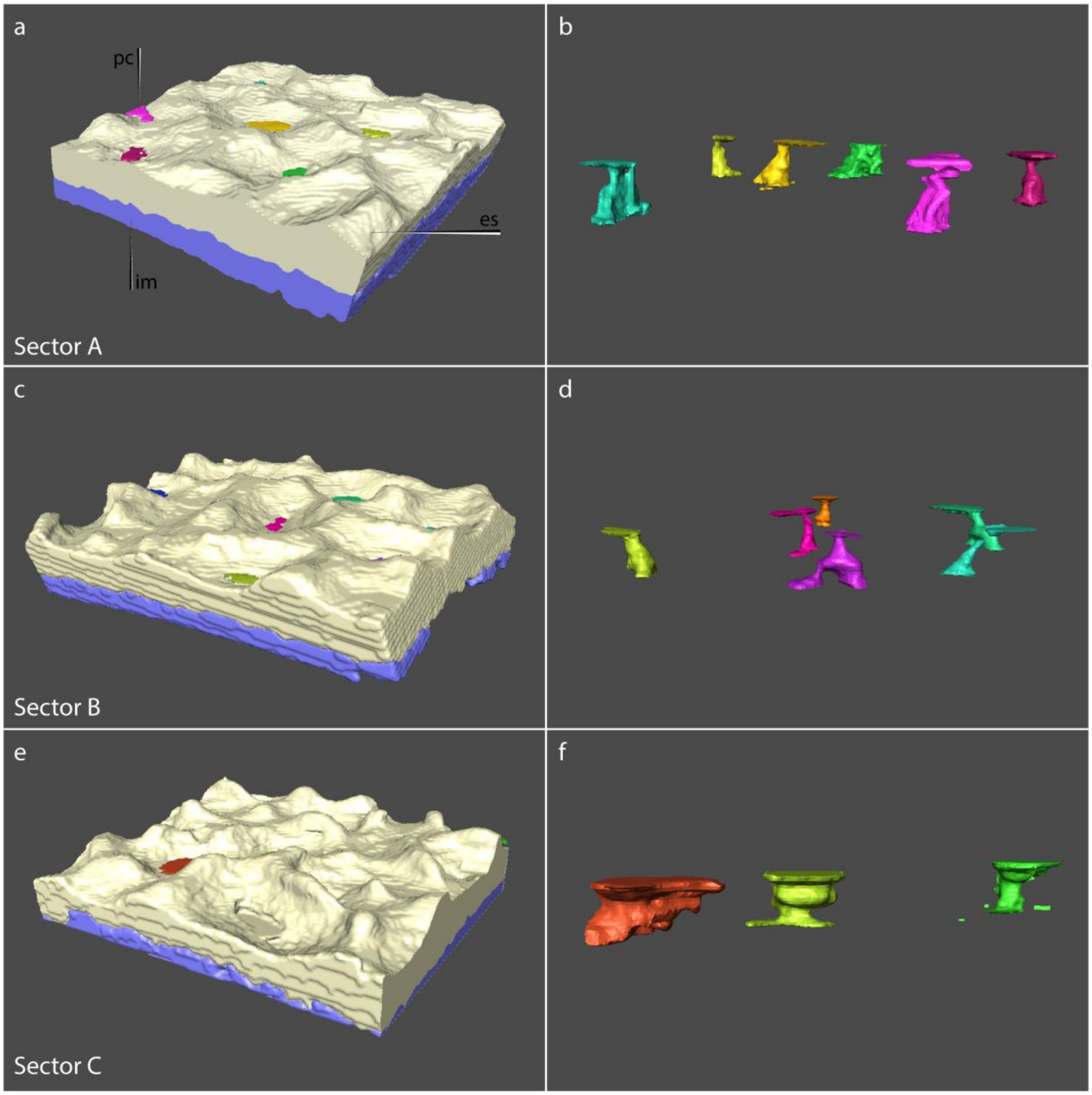

Supplementary Figure S19. Eggshell CY3. (a, b) Sector D. (c, d) Sector E. (e, f) Sector F. Note that some pore canals have extremely wide external ends. As the pore canals get thinner outwards, we included a few outer slices to be sure that all the pore slices were recorded. Abbreviations: es, eggshell; im, inner shell membrane; pc, pore canal. Each sector measures ~9 mm^2.

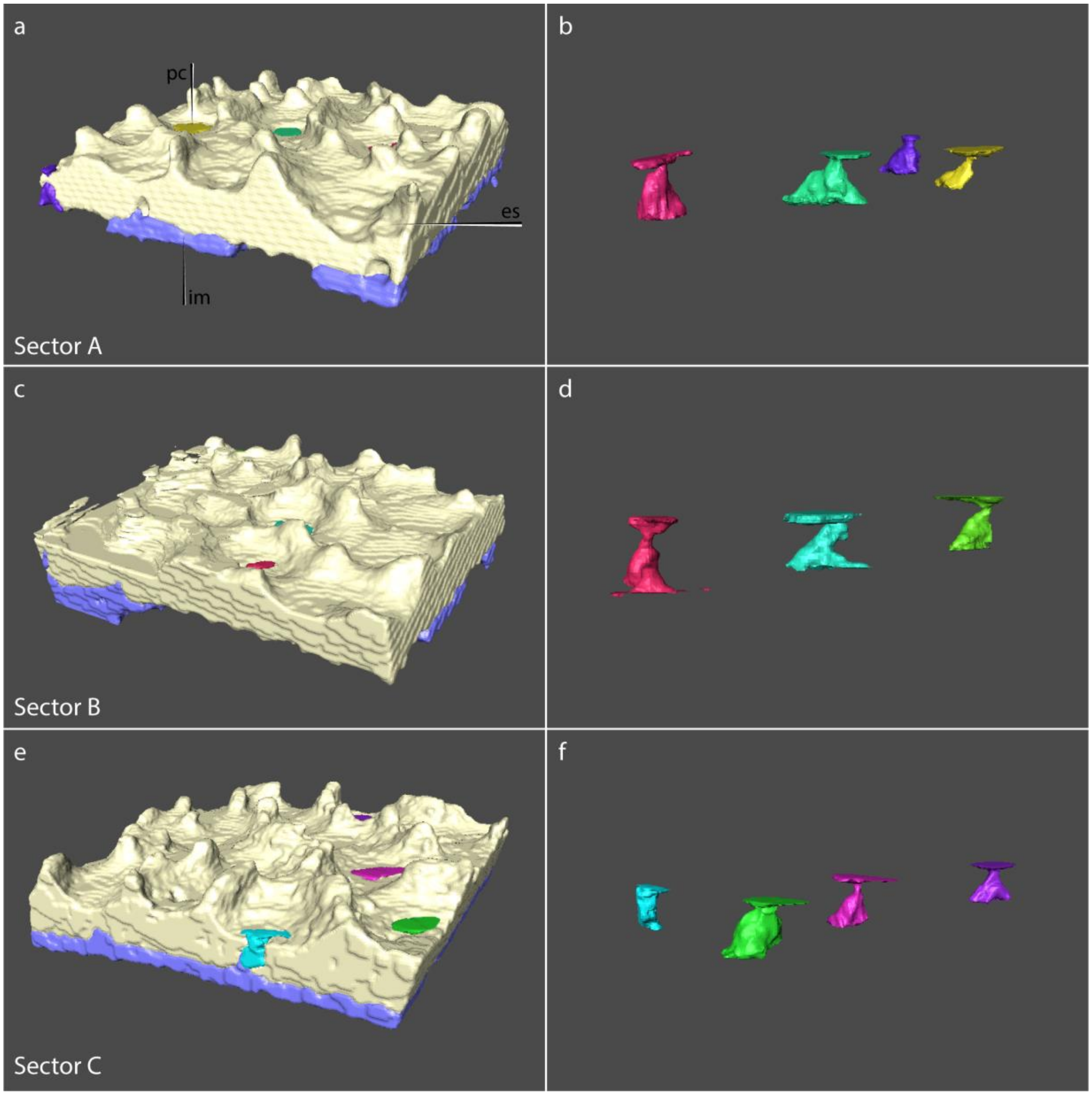

Supplementary Figure S20. Eggshell CY4. (a, b) Sector A. (c, d) Sector B. (e, f) Sector C. Note that some pore canals have extremely wide external ends. As the pore canals get thinner outwards, we included a few outer slices to be sure that all the pore slices were recorded. Abbreviations: es, eggshell; im, inner shell membrane; pc, pore canal. Each sector measures ~9 mm^2.

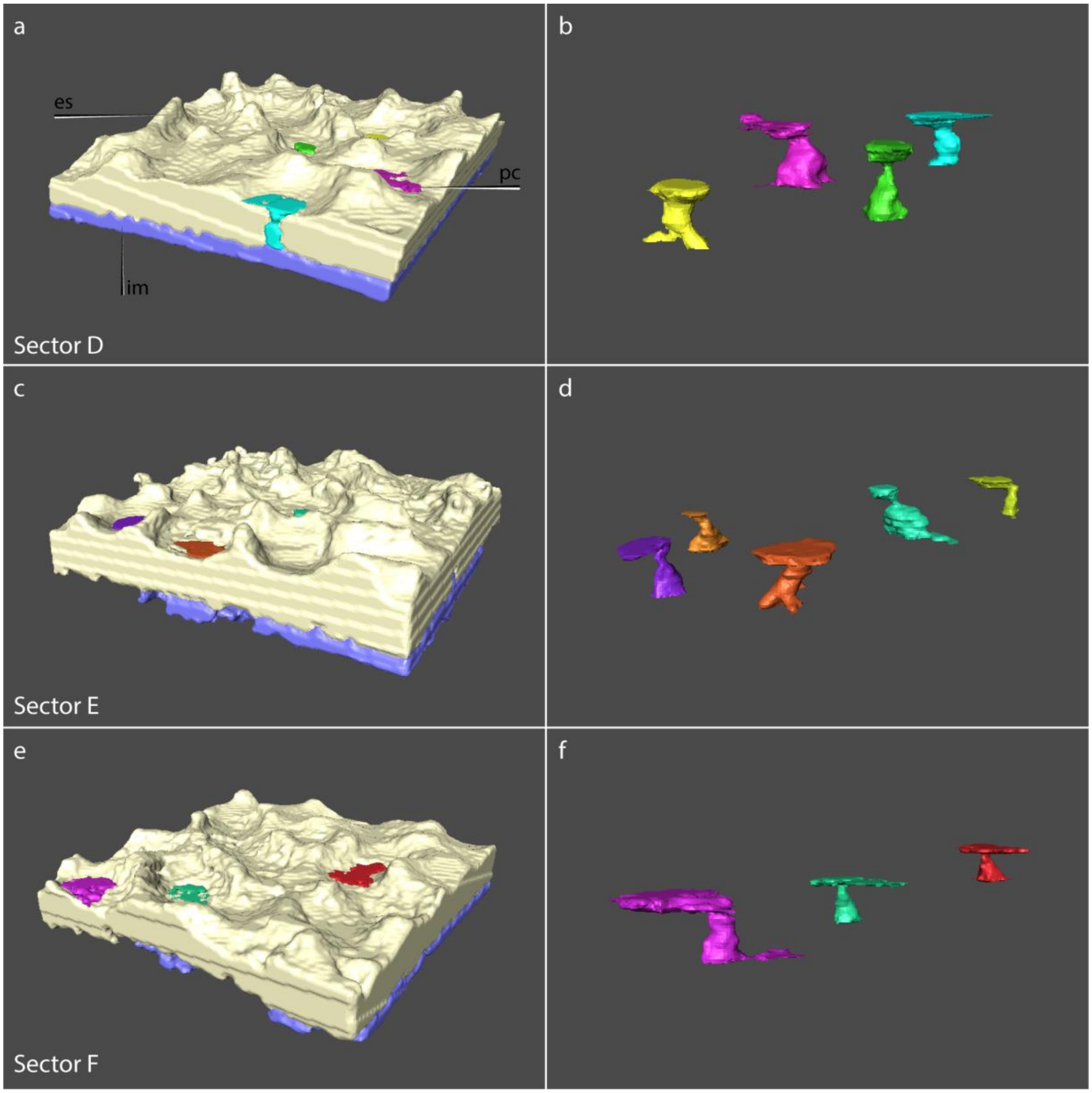

Supplementary Figure S21. Eggshell CY4. (a, b) Sector D. (c, d) Sector E. (e, f) Sector F. Note that some pore canals have extremely wide external ends. As the pore canals get thinner outwards, we included a few outer slices to be sure that all the pore slices were recorded. Abbreviations: es, eggshell; im, inner shell membrane; pc, pore canal. Each sector measures ~9 mm^2.

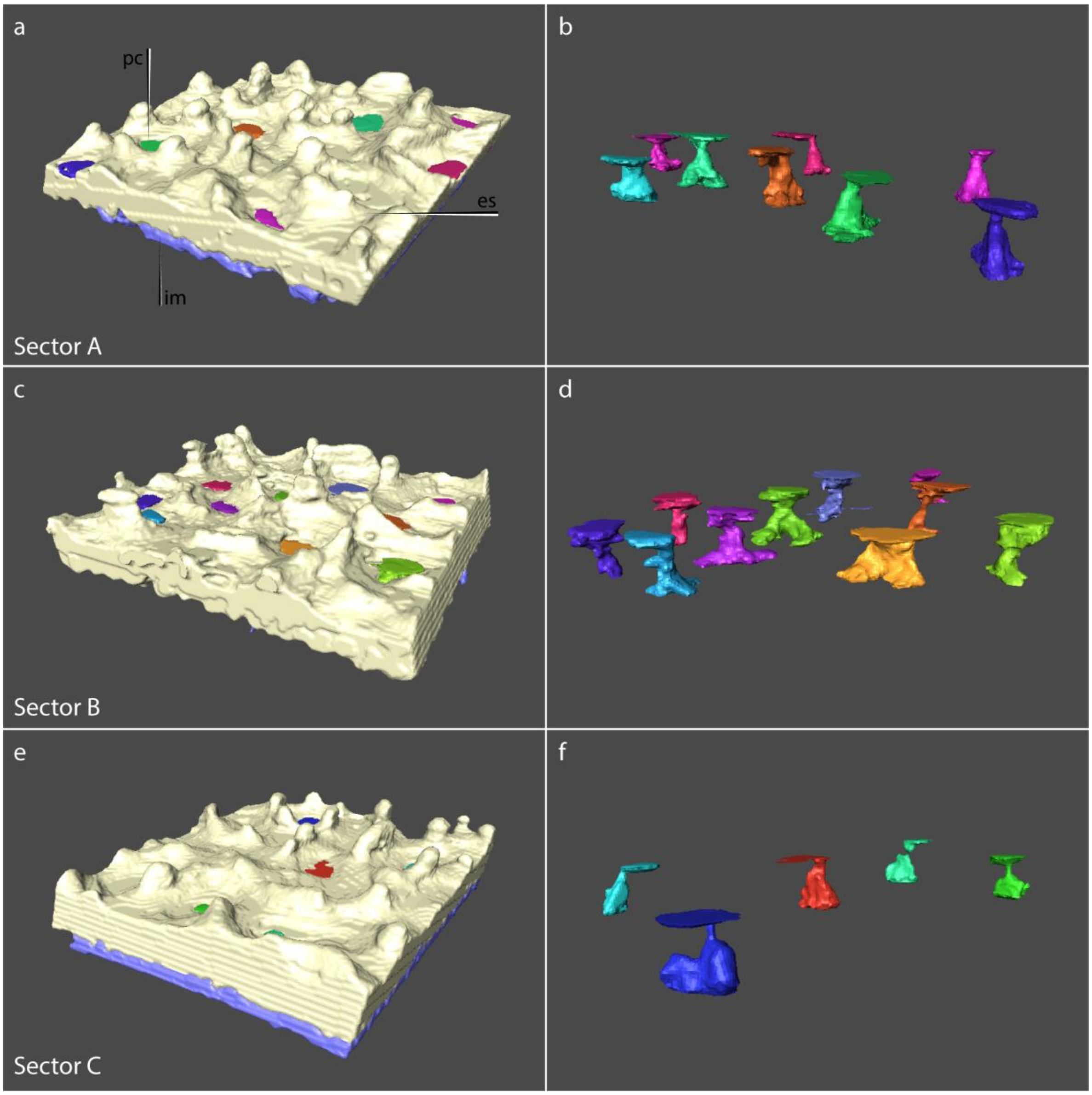

Supplementary Figure S22. Eggshell CY5. (a, b) Sector A. (c, d) Sector B. (e, f) Sector C. Note that some pore canals have extremely wide external ends. As the pore canals get thinner outwards, we included a few outer slices to be sure that all the pore slices were recorded. Abbreviations: es, eggshell; im, inner shell membrane; pc, pore canal. Each sector measures ~9 mm^2.

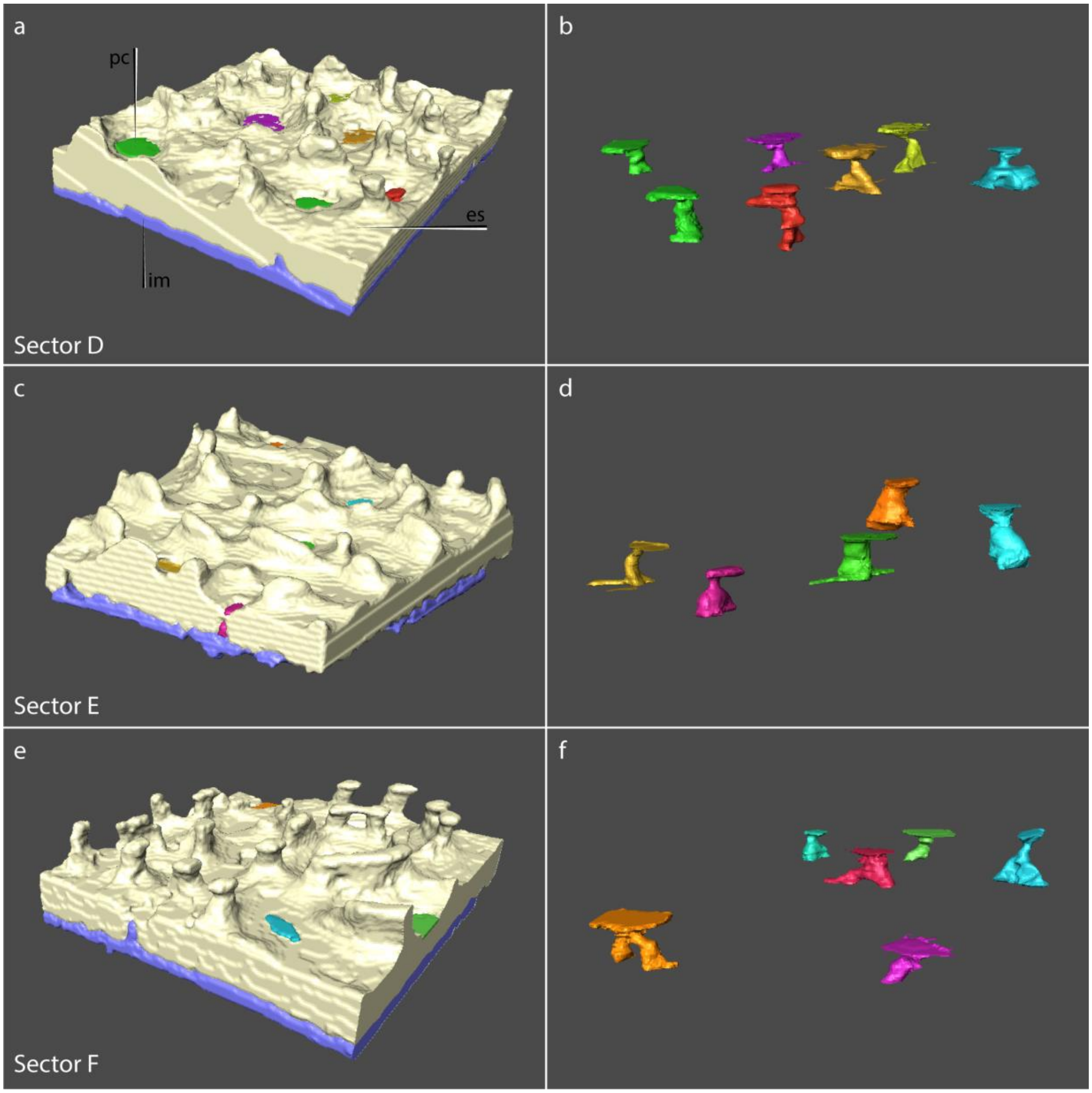

Supplementary Figure S23. Eggshell CY5. (a, b) Sector D. (c, d) Sector E. (e, f) Sector F. Note that some pore canals have extremely wide external ends. As the pore canals get thinner outwards, we included a few outer slices to be sure that all the pore slices were recorded. Abbreviations: es, eggshell; im, inner shell membrane; pc, pore canal. Each sector measures ~9 mm^2.

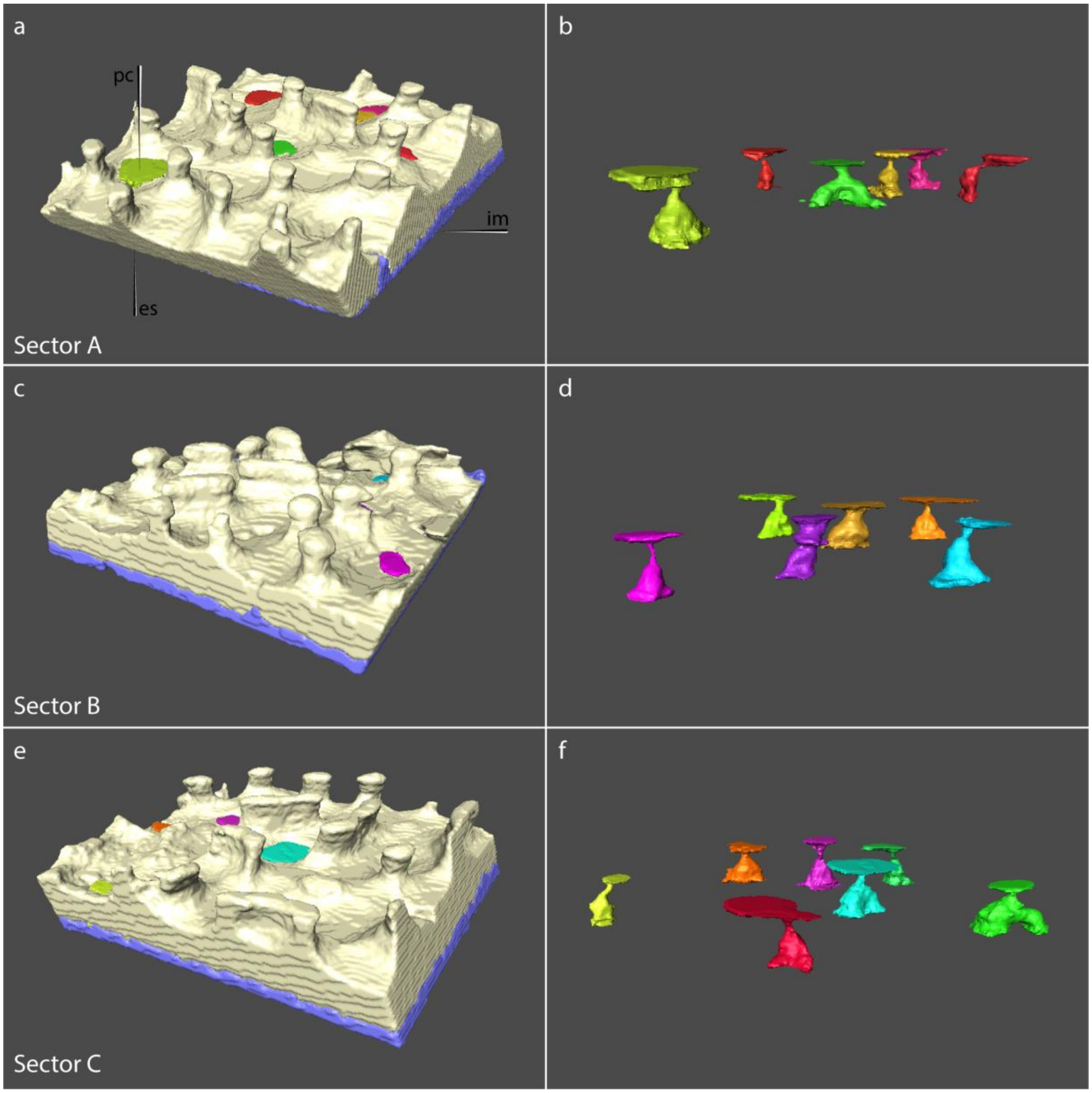

Supplementary Figure S24. Eggshell CY6. (a, b) Sector A. (c, d) Sector B. (e, f) Sector C. Note that some pore canals have extremely wide external ends. As the pore canals get thinner outwards, we included a few outer slices to be sure that all the pore slices were recorded. Abbreviations: es, eggshell; im, inner shell membrane; pc, pore canal. Each sector measures ~9 mm^2.

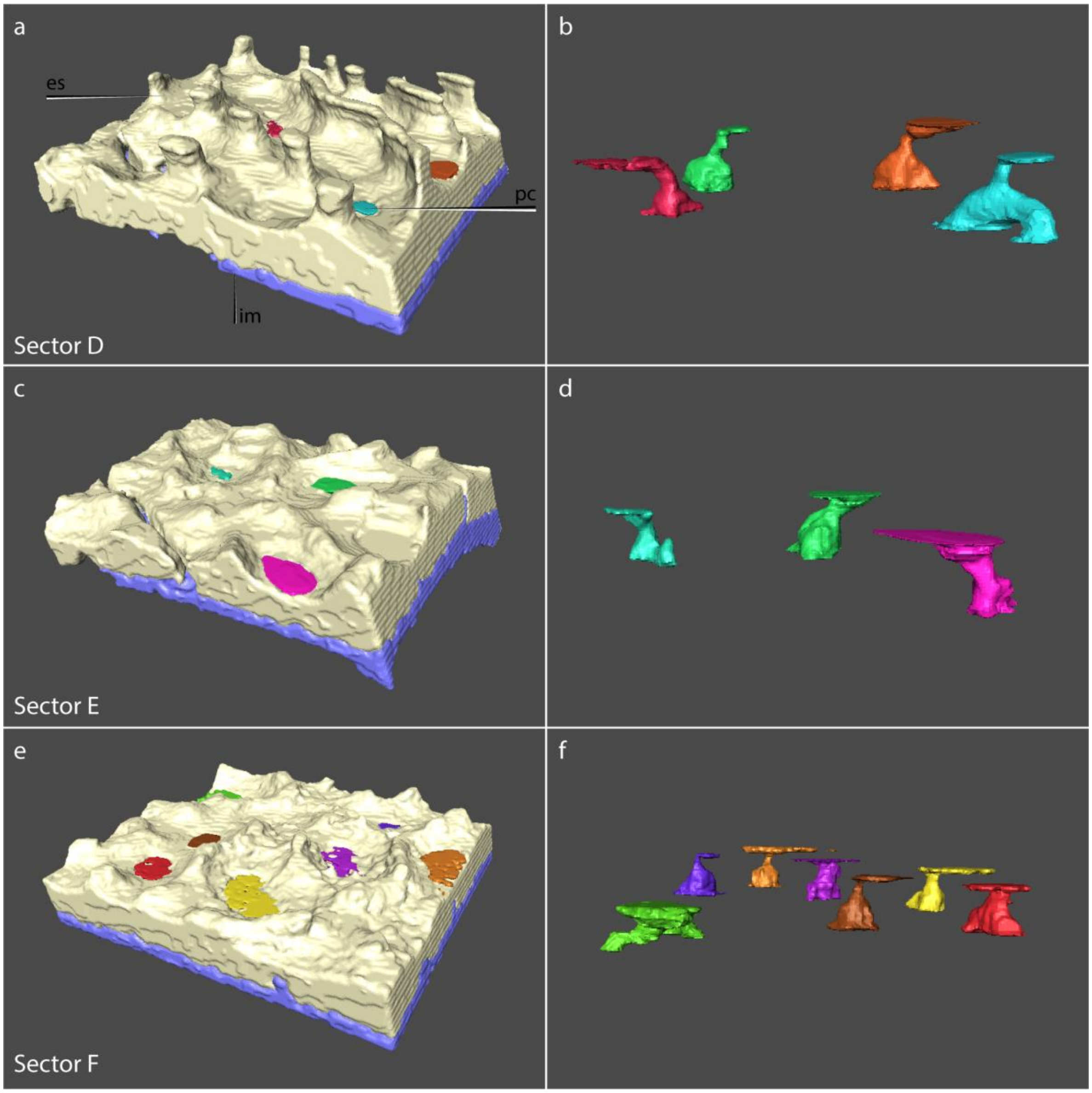

Supplementary Figure S25. Eggshell CY6. (a, b) Sector D. (c, d) Sector E. (e, f) Sector F. Note that some pore canals have extremely wide external ends. As the pore canals get thinner outwards, we included a few outer slices to be sure that all the pore slices were recorded. Abbreviations: es, eggshell; im, inner shell membrane; pc, pore canal. Each sector measures ~9 mm^2.

Eggshell measurements

Measurements regarding eggshell thickness, ornamentation thickness, pore size and density are summarized in the following Supplementary Table S3.

		Eggshell thickness	Compact layer thickness	Pores sampled	Pore density	Pore volume
Unit		µm	µm	pores	pores/cm^2	mm^3
C. latirostris	CL1	597.49 (3.56)	339.20 (6.54)	28	44.44	8.11e-03 (6.17e-04)
	CL2	660.96 (5.19)	328.81 (4.14)	29	46.03	6.13e-03 (5.84e-04)
	CL3	692.09 (4.68)	306.40 (5.66)	28	44.44	8.91e-03 (5.93e-04)
	CL4	662.53 (3.51)	290.62 (3.66)	28	44.44	6.10e-03 (5.65e-04)
	CL5	643.13 (3.55)	264.36 (4.59)	28	44.44	7.92e-03 (7.72e-04)
	CL6	680.28 (2.99)	292.51 (4.02)	39	61.90	9.31e-03 (4.91e-04)
C. yacare	CY1	634.19 (12.09)	258.25 (7.29)	38	60.32	5.61e-03 (4.67e-04)
	CY2	718.04 (5.83)	236.51 (4.85)	32	50.79	5.83e-03 (3.88e-04)
	CY3	474.29 (9.18)	237.78 (5.92)	32	50.79	5.85e-03 (6.33e-04)
	CY4	569.49 (9.40)	258.49 (5.04)	23	36.51	5.37e-03 (4.98e-04)
	CY5	652.71 (6.32)	238.04 (4.43)	41	65.08	6.21e-03 (5.02e-04)
	CY6	658.50 (18.28)	247.50 (3.70)	33	52.38	7.85e-03 (4.70e-04)

Supplementary Table S2. Properties of the eggshells measured in the present study. Values are means and the numbers in parentheses correspond to SE. We took 30 measurements for the thicknesses of the eggshell and the compact layer of each fragment.

Pore cross-sectional area and water vapor conductance (G_{H2O})

After the manual segmentation of all pore canals in each sample, we carried out a label analysis to obtain the pores' cross-sectional area at each slice (Supplementary Table S3, S4). Then, these data were used to calculate the resistance of each pore segment (R_S), which add up to obtain the total pore resistance (R) and its reciprocal, the G_{H2O} (Supplementary Table S3, S4).

Supplementary Table S3. Pore canal cross-sectional area and single pore G_{H2O} in *C. latirostris*. Includes results from label analysis (cross-sectional area) and the calculated single pore R for each slice. G_{H2O} was calculated as: G_{H2O}=1/R. Each slice is 13.93 µm thick. Differences in pore length are associated to local variations in shell thickness.

Supplementary Table S4. Pore canal cross-sectional area and single pore G_{H2O} in *C. yacere*. Includes results from label analysis (cross-sectional area) and the calculated single pore R for each slice. G_{H2O} was calculated as: G_{H2O}=1/R. Each slice is 13.93 µm thick. Differences in pore length are associated to local variations in shell thickness.
